# The mitotic exit mediated by small GTPase Tem1 is essential for the pathogenicity of *Fusarium graminearum*

**DOI:** 10.1371/journal.ppat.1011255

**Published:** 2023-03-16

**Authors:** Pengfei Miao, Xuzhao Mao, Shuang Chen, Yakubu Saddeeq Abubakar, Yulong Li, Wenhui Zheng, Jie Zhou, Zonghua Wang, Huawei Zheng

**Affiliations:** 1 Fujian Key Laboratory on Conservation and Sustainable Utilization of Marine Biodiversity, Fuzhou Institute of Oceanography, College of Geography and Oceanography, Minjiang University, Fuzhou, China; 2 State Key Laboratory of Ecological Pest Control for Fujian and Taiwan Crops, Fujian Agriculture and Forestry University, Fuzhou, China; 3 Department of Biochemistry, Faculty of Life Sciences, Ahmadu Bello University, Zaria, Nigeria; Purdue University, UNITED STATES

## Abstract

The mitotic exit is a key step in cell cycle, but the mechanism of mitotic exit network in the wheat head blight fungus *Fusarium graminearum* remains unclear. *F. graminearum* infects wheat spikelets and colonizes the entire head by growing through the rachis node at the bottom of each spikelet. In this study, we found that a small GTPase FgTem1 plays an important role in *F. graminearum* pathogenicity and functions in regulating the formation of infection structures and invasive hyphal growth on wheat spikelets and wheat coleoptiles, but plays only little roles in vegetative growth and conidiation of the phytopathogen. FgTem1 localizes to both the inner nuclear periphery and the spindle pole bodies, and negatively regulates mitotic exit in *F. graminearum*. Furthermore, the regulatory mechanisms of FgTem1 have been further investigated by high-throughput co-immunoprecipitation and genetic strategies. The septins FgCdc10 and FgCdc11 were demonstrated to interact with the dominant negative form of FgTem1, and FgCdc11 was found to regulate the localization of FgTem1. The cell cycle arrest protein FgBub2-FgBfa1 complex was shown to act as the GTPase-activating protein (GAP) for FgTem1. We further demonstrated that a direct interaction exists between FgBub2 and FgBfa1 which crucially promotes conidiation, pathogenicity and DON production, and negatively regulates septum formation and nuclear division in *F. graminearum*. Deletion of *FgBUB2* and *FgBFA1* genes caused fewer perithecia and immature asci formations, and dramatically down-regulated trichothecene biosynthesis (TRI) gene expressions. Double deletion of *FgBUB2/FgBFA1* genes showed that *FgBUB2* and *FgBFA1* have little functional redundancy in *F. graminearum*. In summary, we systemically demonstrated that FgTem1 and its GAP FgBub2-FgBfa1 complex are required for fungal development and pathogenicity in *F. graminearum*.

## Author summary

Cell cycle is controlled by numerous mechanisms ensuring correct cell division. One fundamental question in the cell cycle is the mitotic exit and entry into the next cycle, but the mechanism of mitotic exit network in the wheat head blight fungus *Fusarium graminearum* remains unclear. In this study, we identified a small GTPase FgTem1 to be involved in mitotic exit in this fungus and demonstrated that this protein is required for the fungal pathogenicity and functions in regulating the infection structures formation and invasive hyphal growth on wheat spikelets and wheat coleoptiles. Furthermore, the regulatory mechanisms of FgTem1 have been further investigated. To the best of our knowledge, this work is the most comprehensive genetic dissection of Tem1 in filamentous fungal pathogens. Our results unveil a key link between mitotic exit network and the pathogenicity of the phytopathogen. These findings provide a novel molecular basis for the design and development of chemicals that prevent phytopathogen infection.

## Introduction

*F. graminearum* is rated among the top 10 economically important fungal pathogens, and causes the devastating head blight disease in wheat and other cereal crops [1–4]. The fungus produces sexual fruiting bodies (perithecia) under conducive climatic conditions (e.g. high humidity), the ascospores released from the perithecia serve as the primary propagules that infect cereal heads at the flowering stages [5]. To initiate infection, the head-blight fungus gains access into the host cells through natural openings, including the stomata [6], before colonizing subsequent host cells and tissues through hyphal elongation [7]. Furthermore, it infects spikelets and colonizes the entire wheat head by growing through the rachis node at the bottom of each spikelet [8,9]. Besides physical destructions, *F. graminearum* also contaminates cereal grains with carcinogenic mycotoxins such as deoxynivalenol (DON). DON also serves as a virulence factor that suppresses the biosynthesis of some defense related proteins in the host cell [9,10]. In addition to trichothecene cluster proteins [11], septins, Rho, FgBud3, FgCdc14 and myosin have equally been shown to positively regulate pathogenesis and DON production in *F. graminearum* [12–19]. These gene products are also required for cell division in *F. graminearum*, suggesting a likely correlation between cell cycle progression and proper pathophysiological development of fungi.

In eukaryotic cells, cell cycle progresses through two distinct phases, namely interphase and mitotic (M) phases. The M phase comprises mitosis and cytokinesis [20]. While mitosis facilitates the segregation of sister chromatids into two identical nascent nuclei, cytokinesis mediates the partitioning and physical division of the cytoplasm of the parent cell into two daughter cells [20]. In fungi, cytokinesis is a three-stage process which involves selection of division site, orderly assembly of protein complexes and institution of the dynamic events required to initiate constriction of the contractile ring and septum formation [21]. The coupling of nuclear division (mitosis) with cytokinesis involves the actions of two elaborate network systems known as the mitotic exit network (MEN) and septation initiation network (SIN) [22].

The MEN is a signaling pathway that mediates mitosis exit and the entry into cytokinesis and positively promote cell division in budding yeast [23]. The MEN pathway resembles a Ras-like GTPase signaling cascade and it comprises the small GTPase Tem1, guanine-nucleotide exchange factor Lte1, the two-component GTPase activating protein Bub2-Bfa1, three protein kinases (Cdc5, Cdc15, Dbf2), the Dbf2-associated factor Mob1 and a scaffold protein Nud1 [24]. Tem1 controls the essential mitotic processes including spindle assembly, spindle orientation and initiation of DNA damage repair in conjunction with Bfa1 and Bub2 [22,25]. Studies in budding yeast provided insights into the common cell cycle control mechanisms in eukaryotes. The Bub2 pathway monitors defects in cytoplasmic microtubule structures [26], and Bfa1 and Bub2 may function as a universal checkpoint in response to various defects such as DNA damage or spindle misorientation [27]. Negative regulation of mitotic exit by Bfa1 and Bub2 is likely to be important to prevent Tem1-mediated mitotic exit before completion of anaphase B [22].

*Schizosaccharomyces pombe* and *Aspergillus nidulans* have a signaling pathway analogous to MEN, called the SIN pathway, and the primary role of this network is to regulate septation rather than mitotic exit [28,29], where Bub2 has been demonstrated in the fungi to regulate septum formation rather than mitotic exit [30]. Deletion of *CoBUB2* resulted in earlier onset of nuclear division and decreased the time for G_1_/S cell cycle progression during appressorium development in the cucumber anthracnose fungus *Colletotrichum orbiculare* [31]. In *Colletotrichum higginsianum* and *Magnaporthe oryzae, BUB2* regulates the G_1_/S transition, septum formation and nuclear division [32]. Tem1 is the down-stream target of Bub2-Bfa1. In yeast, Tem1 is involved in the mitotic exit network and plays a critical role in regulating cell division. In *S. pombe*, the Tem1 homolog Spg1 essentially controls the onset of septum formation. Another Tem1 homolog Ras3 regulates nuclear envelope breakdown rather than mitotic exit or septum formation in the basidiomycete *Ustilago maydis* [33]. These previous studies suggest the functional divergence of Tem1, Bub2 and Bfa1 in filamentous fungi.

Orthologs of Tem1 are well conserved in plant pathogenic fungi. However, the role of Tem1 homologs remain poorly understood in phytopathogens and systematic studies on its roles especially in relation to pathogenesis are required. In this study, we systematically investigate the function of FgTem1 in the development and pathogenesis of the wheat pathogen *F. graminearum*. Our results show that FgTem1 plays relatively weak roles in the fungal vegetative growth and conidiation, but it is crucial for pathogenicity, and negatively regulates nuclear division. Furthermore, FgBub2-FgBfa1 complex is found to function as a GAP for FgTem1, and negatively regulates septum formation and nuclear division, and is important for conidiation, sexual reproduction, DON production and pathogenicity in *F. graminearum*.

## Results

### Phylogenetic characterization of Tem1 orthologs

To identify the FgTem1 protein in *F. graminearum* for subsequent functional characterization of the protein in the head-blight fungus, the amino acid sequence of *S. cerevisiae* Tem1 (ScTem1, QHB10674.1) was retrieved from the NCBI database and used to conduct a BLAST-search in fungal and oomycetes genome resources database (http://www.fungidb.org/fungidb/showApplication.do). Results obtained from the BLAST-search identified the ScTem1 ortholog FgTem1 in the head-blight fungus encoded by FGSG_17139 gene. Detailed sequence analysis showed that the putative FgTem1 identified in *F. graminearum* shares 57% identity with the ScTem1 protein. The FgTem1 sequence query covered 73% of the total length of ScTem1. Corresponding results derived from the phylogenetic analysis showed that Tem1 is phylogenetically conserved in plant pathogenic fungi, including *Fusarium* and *Magnaporthe oryzae*, but distant from Tem1 in *Arabidopsis thaliana* (S1 Fig).

### FgTem1 contributes to vegetative growth and conidiation of *F. graminearum*

**Fig 1:**
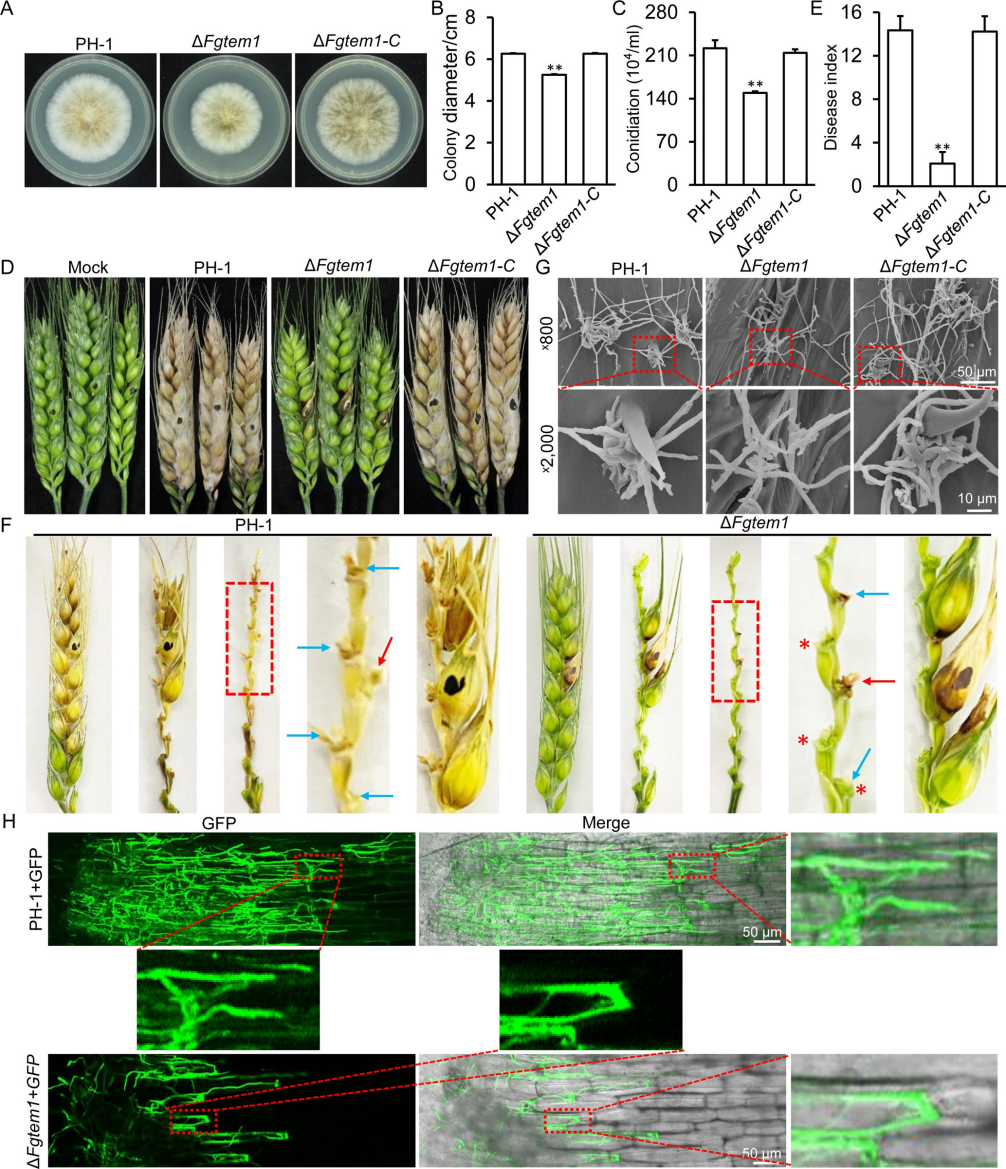
FgTem1 is critically required for the pathogenicity of *F. graminearum*. (A-B) shows the colonies and colony diameters of the wild type PH-1, *FgTEM1* deletion mutant (Δ*Fgtem1*) and complemented strain (Δ*Fgtem1*-C) on complete medium (CM) agar plates after 3 days of incubation. Two-tailed Student *t*-test was used for paired comparison of the colony diameter between Δ*Fgtem1* and PH-1. (**P < 0.01). (C) Conidiation of PH-1, Δ*Fgtem1* and Δ*Fgtem1*-C in CMC media after 3 days. Two-tailed Student *t*-test was used for paired comparison of the conidiation between Δ*Fgtem1* and PH-1. (**P < 0.01). (D) Pathogenicity of PH-1, Δ*Fgtem1* and Δ*Fgtem1*-C in wheat heads. The infected wheat heads were examined at 14 days after inoculation. (E) The disease indexes of the indicated strains in wheat heads. Three independent biological replicates were involved for each strain. Two-tailed Student *t*-test was used for paired comparison of the conidiation between Δ*Fgtem1* and PH-1. (**P < 0.01). (F) Pathogenicity of PH-1, Δ*Fgtem1* and Δ*Fgtem1*-C in wheat rachis nodes. Infected wheat rachises were examined at 14 days after inoculation. Red arrowheads indicate the inoculated wheat heads and basal rachis nodes, blue arrowheads indicate the basal rachis nodes of the symptomatic wheat heads, red asterisks indicate the rachis nodes of non- or symptomatic wheat heads. Shown is a result of three independent experiments. (G) Infection structures formed by the indicated strains on wheat lemma at 2 dpi were examined by SEM under ×800 and ×2,000 magnification. The representative micrographs show the defect in infection structure formation in the Δ*Fgtem1* mutant. (H) Confocal images of the PH-1+GFP and Δ*Fgtem1*+GFP strains of *F. graminearum* growing inside the wheat coleoptiles for 24 h.

To evaluate the contributions of FgTem1 to both physiological and pathological developments of *F. graminearum*, we used targeted gene replacement strategies to delete the *FgTEM1* gene in the fungus, and obtained four independent *FgTEM1* gene defective strains (Δ*Fgtem1*-1, Δ*Fgtem1*-3, Δ*Fgtem1*-33 and Δ*Fgtem1*-34), the gene deletions were further confirmed by Southern blotting (S2A Fig). To complement the defects associated with the Δ*Fgtem1* mutant, we further generated FgTem1-GFP fusion construct under the control of *FgTEM1* native promoter and re-introduced the construct into the protoplasts of the Δ*Fgtem1* strain and obtained Δ*Fgtem1*-C (complementation) strain. PH-1, Δ*Fgtem1*, and the Δ*Fgtem1*-C strains were subjected to vegetative growth and conidiation assessment assays. The phenotypic assessment results showed a slight reduction in the vegetative growths of the Δ*Fgtem1* strains relative to the wild type and the Δ*Fgtem1*-C strain (Fig 1A and 1B). Furthermore, there was a slight reduction in the number of conidia produced by the Δ*Fgtem1* mutant compared to the PH-1 and Δ*Fgtem1*-C strains (Fig 1C). These results indicate that FgTem1 only plays slight roles in the normal vegetative growth and conidiation of *F. graminearum*.

### FgTem1 is required for the pathogenicity of *F. graminearum*

To assay for the infection ability of the Δ*Fgtem1* mutant on flowering wheat heads, we inoculated different flowering wheat heads with Δ*Fgtem1* mutant as well as the PH-1 and Δ*Fgtem1*-C strains as controls, respectively, and incubated them under a moist-condition for 14 days. The results of the comparative infection assays reveal that the head blight symptoms caused by the Δ*Fgtem1* mutant spread to the nearby spikelets at a much slower rate than the controls under the same conditions (Fig 1D and 1E). In wheat, *F. graminearum* infects spikelets and colonizes the entire head by growing through the rachis node at the bottom of each spikelet [8,9]. Therefore, we checked the wheat rachis node tissues of these inoculated wheat spikelets and found that the Δ*Fgtem1* mutants were unable to spread to adjacent rachis nodes compared to the wild type (Fig 1F). The observed head blight symptoms developed by the mutant could be due to its direct contact with the inoculated wheat heads since the red asterisked heads (rachis nodes) do not show the symptom (Fig 1F), suggesting that FgTem1 is critically indispensable for the invasive hyphal growth of *F. graminearum* in wheat rachis node. When examined by scanning electron microscopy (SEM), the *FgTEM1* deletion mutant formed smaller infection structures (cushions) on wheat lemma than the wild type PH-1 and complementation strain Δ*Fgtem1*-C (Fig 1G). In addition, FgTem1 is also important for the virulence on corn silk (S3 Fig). Furthermore, to evaluate the roles of *FgTEM1* disruption on the invasion process in planta at the cellular level, a GFP plasmid was transformed into the PH-1 and Δ*Fgtem1* mutant respectively, resulting in PH-1+GFP and Δ*Fgtem1*+GFP strains. As shown in Fig 1H, microscopic examination of hyphal fronts of the PH-1+GFP and Δ*Fgtem1*+GFP strains growing in coleoptile cells showed that the invasive hyphae of the Δ*Fgtem1*+GFP exhibited limited growth in the intercellular space of the wheat coleoptiles, while the PH-1 showed normal growth in wheat coleoptile cells. Taken together, these results suggest that the significant reduction in virulence of Δ*Fgtem1* mutant maybe due to the loss of the ability to form infection structures and its compromised invasive hyphal growth in the wheat spikelets and wheat coleoptile cells.

Deoxynivalenol (DON) represents the most characterized virulence factor in *F. graminearum* [9]. Therefore, to ascertain whether FgTem1 contributes to DON production in *F. graminearum*, the PH-1, Δ*Fgtem1* and Δ*Fgtem1*-C strains were cultured in liquid trichothecene biosynthesis induction (TBI) media for DON production and subsequent DON assay. We found that FgTem1 plays a dispensable role in DON production of *F. graminearum* (S4 Fig). In addition, FgTem1 is dispensable for sexual reproduction of *F. graminearum* (S5A and S5B Fig). Taken together, we conclude that FgTem1 is required for the pathogenicity of *F. graminearum*, consistent with the expression level of *FgTEM1* during wheat head infection is higher than that observed from in vitro experiments during sexual development and DON induction conditions (S1 Table).

### FgTem1 negatively regulates nuclear division in *F. graminearum*

**Fig 2:**
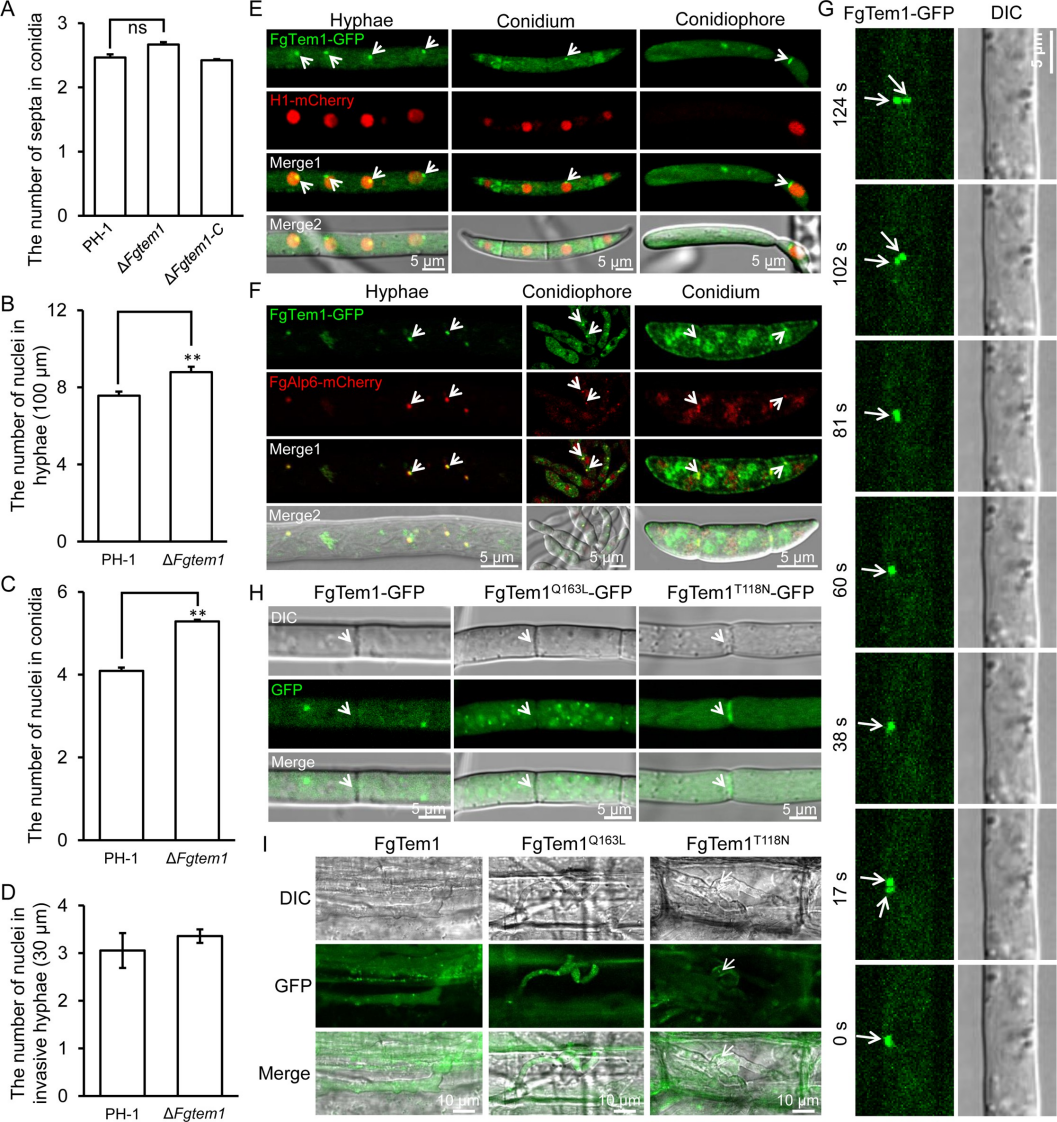
FgTem1 plays an important role in nuclear division and localizes to spindle pole bodies. (A) The number of septa in the conidia of the indicated strains. Two-tailed Student *t*-test was used for paired comparison of the number of septa in the conidia of Δ*Fgtem1* and PH-1. (P = 0.06, ns, no significant difference). (B-C) The number of nuclei in the hyphae (100 μm) and conidia of the indicated strains. (**P < 0.01). (D) The number of nuclei in the invasive hyphae (30 μm) of PH-1 and Δ*Fgtem1* strains. (E) FgTem1-GFP localizes to the nuclear periphery in the hyphae, conidiophore and conidium. H1-mCherry is expressed to mark the nuclei. White arrowheads show the FgTem1-GFP localization. (F) FgTem1-GFP co-localizes with FgAlp6-mCherry in *F. graminearum* hyphae, conidiophores and conidia. FgAlp6-mCherry is a spindle pole body (SPB) marker. White arrowheads show the co-localization. (G) Time-lapse images of FgTem1-GFP, times are indicated in seconds. White arrowheads show the FgTem1-GFP localization. (H) The localization of constitutive activate and dominant negative isoforms of FgTem1-GFP (FgTem1^Q163L^-GFP and FgTem1^T118N^-GFP) in the hyphae. White arrowheads show the septa. (I) The localization of FgTem1-GFP, FgTem1^Q163L^-GFP and FgTem1^T118N^-GFP) in wheat coleoptiles. White arrowheads show the septa.

In yeast, Tem1 is involved in mitotic exit network (MEN) and plays an important role in regulating cell division [34]. Septum formation and nuclear division are crucial indicators of cell division in filamentous fungi [21]. To investigate whether FgTem1 is involved in cell division, we examined the presence of septa in the conidia of the PH-1, Δ*Fgtem1* and Δ*Fgtem1*-C strains harvested from CMC media after 3 days of inoculation. We found a slight and insignificant increase in the number of septa formed in the Δ*Fgtem1* mutant conidia (Fig 2A). Furthermore, we checked whether FgTem1 is involved in nuclear distribution and division in *F. graminearum* by tagging the nuclear marker Histone 1 with GFP (H1-GFP) in the wild type PH-1 and Δ*Fgtem1* mutant, and then examined the nuclear distribution and division by confocal microscopy. Although targeted disruption of *FgTEM1* has no significant effects on nuclear distribution (S6A Fig), we observed that the number of nuclei per 100 μm hyphae and per conidium slightly increased in the Δ*Fgtem1* mutant (Fig 2B and 2C). In addition, the number of nuclei per 30 μm length of the invasive hyphae in the Δ*Fgtem1* mutant was also slightly increased compared with that in the wild type PH-1 (Fig 2D). Taken together, these results indicate that FgTem1 negatively regulates nuclear division in *F. graminearum* and may be involved in MEN.

### The subcellular localization of FgTem1

The localization pattern of Tem1 homologs in plant pathogenic fungi is still unknown. To unveil the subcellular localization of this protein in *F. graminearum*, we subjected the FgTem1-GFP expressing strain (Δ*Fgtem1*-C) to laser confocal microscopy and found that FgTem1-GFP appears as punctate structures in the hyphae, conidiophore and conidia of the fungus (Fig 2E). Since FgTem1 is involved in nuclear division, we co-transformed FgTem1-GFP construct with the nucleus marker H1-RFP into the protoplasts of the wild type PH-1. Microscopic investigations showed that FgTem1-GFP localizes to the nuclear periphery of the fungal hyphae, conidiophores and conidia (Fig 2E). Consistently, a 3-D (three-dimensional) micrograph of the GFP and RFP signals further support these results (S6B and S6C Fig). A previous study in yeast demonstrated that Tem1 protein is associated with spindle pole bodies (SPB) [35]. To check this in *F. graminearum*, we tagged the SPB marker FgAlp6 (FGSG_09910, an ortholog of *M. oryzae* Alp6) [36] with mCherry and co-transformed it with FgTem1-GFP construct into the Δ*Fgtem1* mutant background and observed the positive transformant under a confocal microscope. As shown in Fig 2F, FgTem1-GFP co-localizes with FgAlp6-mCherry in the fungal hyphae, conidiophores and conidia, suggesting that FgTem1 localizes to the SPB at different developmental stages of *F. graminearum*. In addition, time-lapse images and their corresponding video (Fig 2G and S1 Video) showed that FgTem1 is dynamic and varies between individual poles in the fungal hyphae. Furthermore, in line with some previous studies [31,37], we generated dominant negative (FgTem1^T118N^-GFP) and constitutively activate (FgTem1^Q163L^-GFP) isoforms of FgTem1. As shown in S7A and S7B Fig, the vegetative growth of FgTem1^Q163L^ and FgTem1^T118N^ strains are slightly reduced compared to those of the wild type PH-1. We further found that the dominant negative isoform of FgTem1 (FgTem1^T118N^-GFP) in the wild type PH-1 lost the SPB localization and eventually mislocalizes to the septa and cytoplasm of the fungal hyphae, unlike the normal localization pattern observed for FgTem1-GFP as well as its constitutively activate isoform (FgTem1^Q163L^-GFP) in the wild type PH-1 (Fig 2H). As shown in Fig 2I, the localizations of FgTem1-GFP, FgTem1^Q163L^-GFP and FgTem1^T118N^-GFP in the invasive hyphae are similar to those in vegetative hyphae. Taken together, these results suggest that FgTem1 localizes to SPB at different developmental stages.

### The septins FgCdc10 and FgCdc11 directly interact with the dominant negative isoform of FgTem1

In an attempt to investigate the regulatory mechanisms of FgTem1 functions, FgTem1-GFP was immunoprecipitated using a GFP-Trap_A kit to identify the interacting proteins of FgTem1. The proteins bound to the GFP-Trap beads were eluted, digested with trypsin and subjected to LC-MS/MS analysis. Interestingly, cell division control (Cdc) proteins 3, 10, 11 and FGSG_05337 were captured among the potential FgTem1-GFP-pull-down interacting proteins (Table 1). FgCdc3, 10 and 11 were reported to belong to the septin family and are involved in nuclear division, morphogenesis and pathogenicity of *F. graminearum* [17]. FGSG_05337 is an uncharacterized Cdc protein; unfortunately, we could not obtain FGSG_05337 gene deletion mutants after several attempts, suggesting that deletion of FGSG_05337 is lethal.

**Table 1:** List of identified FgTem1-interacting proteins.

Gene ID	Description
FGSG_17139	Septum-promoting GTP-binding protein 1 (FgTem1)
FGSG_01065	Related to cell cycle arrest protein Bub2 (FgBub2)
FGSG_05315	Cell division control protein 3 (FgCdc3)
FGSG_06035	Cell division control protein 10 (FgCdc10)
FGSG_09421	Cell division control protein 11 (FgCdc11)
FGSG_05537	Cell division cycle

**Fig 3:**
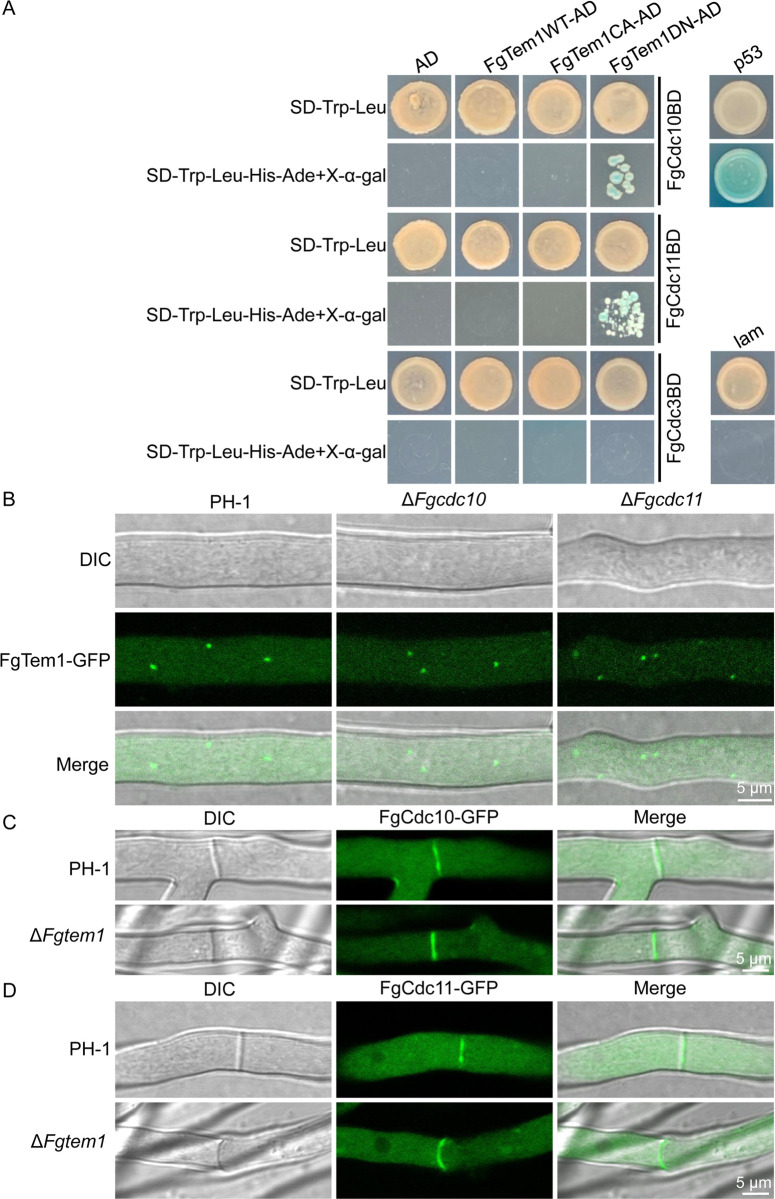
The relationship of FgTem1 with septins in *F. graminearum*. (A) Yeast two-hybrid assay showing the interaction of FgTem1 (FgTem1WT, FgTem1CA and FgTem1DN) with septins (FgCdc3, FgCdc10 and FgCdc11). The prey and bait constructs were assayed for growth on SD-Trp-Leu and SD-Trp-Leu-His-Ade+X-α-gal plates. p53 (pGADT7-T and pGBKT7-p53) and lam (pGADT7-T and pGBKT7-lam) were used as positive and negative controls, respectively. AD (pGADT7) and FgCdc3-BD, FgCdc10-BD, FgCdc11-BD were used as self-activated controls. (B) FgCdc11 is required for the localization of FgTem1-GFP while FgCdc10 is not required. (C-D) FgTem1 is not important for the localization of FgCdc10-GFP and FgCdc11-GFP.

The Ras-like GTPase Tem1 is considered activated in GTP-bound form and inactivated in GDP-bound form [31,38]. Septins were shown to negatively regulate nuclear division in *F. graminearum* [17]. To investigate the relationship between the septins FgCdc3, FgCdc10, FgCdc11 and FgTem1, we performed yeast two-hybrid assays. pFgCdc3BD, pFgCdc10BD, pFgCdc11BD, pFgTem1WT-AD, pFgTem1CA-AD (Q163L, constitutively active form) and pFgTem1DN-AD (T118N, dominant negative form) vectors were constructed, and different combinations of pFgCdc3BD, pFgCdc10BD, pFgCdc11BD with pGADT7(AD), pFgTem1WT-AD, pFgTem1CA-AD and pFgTem1DN-AD vectors were co-transformed into the yeast strain AH109 for the yeast two-hybrid assays. As shown in Fig 3A, we found that FgCdc10 and FgCdc11 specifically interact with the dominant negative form of FgTem1 (FgTem1DN), suggesting that FgCdc10 and FgCdc11 could associate with the dominant negative form of FgTem1. To further unveil the relationship of septins FgCdc10 and FgCdc11 with FgTem1, we transformed FgTem1-GFP vector into the protoplasts of Δ*Fgcdc10* and Δ*Fgcdc11* mutants, respectively. As shown in Fig 3B, the punctate GFP signal of FgTem1 is significantly increased in the Δ*Fgcdc11* mutant compared to that in wild type, suggesting that FgCdc11 is important for the localization of FgTem1 while FgCdc10 is not required. In addition, deletion of *FgTEM1* does not significantly affect the septum localization of FgCdc10-GFP and FgCdc11-GFP (Fig 3C and 3D), suggesting that the septum localization of FgCdc10 and FgCdc11 does not depend on FgTem1 in *F. graminearum*.

### FgBfa1-FgBub2 is a GAP complex for FgTem1

**Fig 4:**
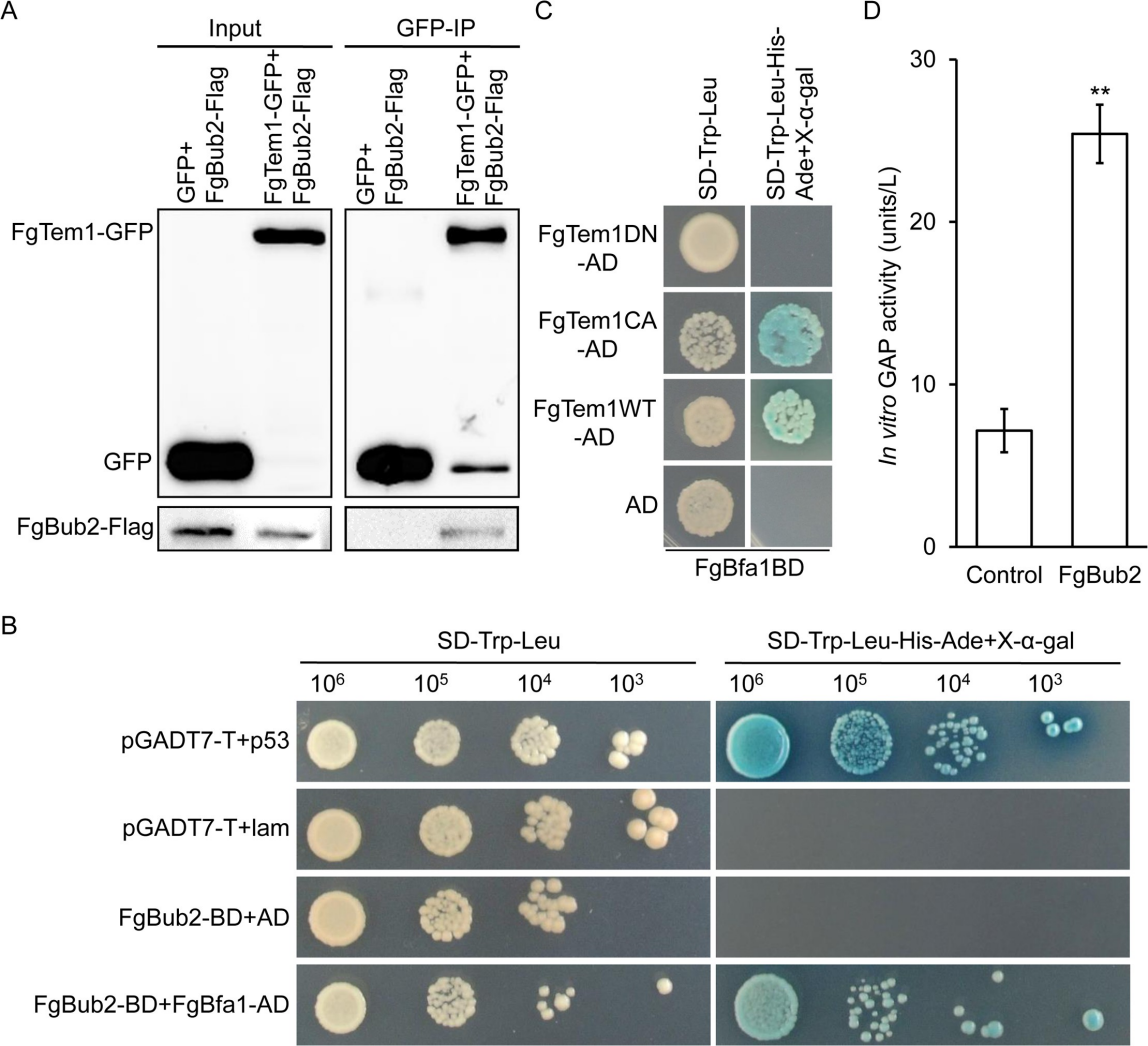
Interacting relationship of FgBub2 and FgBfa1 with FgTem1. (A) Co-IP assay showing positive interaction between FgBub2 and FgTem1. (B) Yeast two-hybrid assay showing positive interaction between FgBub2 and FgBfa1. (C) FgBfa1 directly interacts with FgTem1WT and FgTem1CA (Q163L) but not with FgTem1DN (T118N) in a yeast two-hybrid assay. (D) *In vitro* GAP activity assay of the His-FgBub2. Control: His. One unit is the amount of enzyme that catalyzes the production of 1 μmol free phosphate per minute under the assay conditions. (**P < 0.01).

Since septins were well studied in *F. graminearum*, we looked back to our LC-MS/MS data from the FgTem1-GFP Co-IP assay to screen for more potential FgTem1 interacting proteins. In addition to the cell division control proteins, we identified the cell cycle arrest protein FgBub2 (FGSG_01065) as an FgTem1-associated protein (Table 1). To confirm the interaction, we carried out a co-immunoprecipitation (Co-IP) experiment and the result further verified the positive interaction between FgTem1 and FgBub2 (Fig 4A). Phylogenetic relationships of Bub2 homolog suggest that Bub2 is conserved in plant pathogenic fungi, especially in *Fusarium* and *M. oryzae* (S8A Fig).

Bfa1-Bub2 complex acts as a GAP protein for Tem1 in yeast and *C. orbiculare* [27,31]. We therefore tried to check whether FgBfa1 interacts with FgBub2 to form a complex and whether the heterodimer act as a GAP protein for Tem1 in *F. graminearum*. First, we identified the gene locus for FgBfa1 by using the *S. cerevisiae* Bfa1 amino acid sequence to run a BLAST search where the Bfa1 homolog was eventually identified at FGSG_06242 locus in *F. graminearum* genome. The gene product FgBfa1 shares 32.04% identity with the yeast Bfa1, but the sequence queries cover 10% of their total lengths between FgBfa1 and yeast Bfa1. Phylogenetic analysis of Bfa1 homologs suggests that Bfa1 is also conserved in plant pathogenic fungi (S8B Fig). We further used yeast two-hybrid assay to show that FgBub2 and FgBfa1 directly interact with each other (Fig 4B), suggesting that FgBub2 and FgBfa1 form a heterodimer in *F. graminearum*. To ascertain whether the FgBfa1-FgBub2 complex acts as a GAP for FgTem1 in *F. graminearum*, different combinations of pFgBfa1-BD with pFgTem1WT-AD, pFgTem1CA-AD and pFgTem1DN-AD vectors were respectively co-transformed into the yeast strain AH109 for yeast two-hybrid assays. The results showed that FgBfa1 directly interacts with both FgTem1WT and FgTem1CA; meanwhile, FgBfa1 failed to interact with FgTem1DN (Fig 4C). Furthermore, to determine whether FgBub2 possesses the GAP activity that facilitates the hydrolysis of GTP-bound FgTem1, we performed an *in vitro* GAP activity assay, as shown in Fig 4D, we found that FgBub2 has higher efficiency for hydrolyzing GTP-bound FgTem1 in comparison with the control. Taken together, these results suggest that FgBfa1 and FgBub2 form a complex which likely acts as a GAP for FgTem1 in *F. graminearum*.

### Generation and characterization of *FgBUB2* and *FgBFA1* gene deletion mutants

To understand the physiological functions of FgBub2 and FgBfa1 in *F. graminearum*, we deployed targeted gene replacement strategy to delete *FgBUB2* and *FgBFA1* genes (Fig S2B and S2C) in PH-1 and obtained several positive *FgBUB2* and *FgBFA1* deletion transformants using PCR screening. Further confirmations of targeted replacements of FgBUB2 and FgBFA1 were conducted by subjecting Δ*Fgbub2*-1, Δ*Fgbub2*-2, Δ*Fgbub2*-3, Δ*Fgbfa1*-5, Δ*Fgbfa1*-8, Δ*Fgbfa1*-9 and PH-1 to Southern blot analyses (Fig S2B and S2C). The correct mutants (Δ*Fgbub2*-2, Δ*Fgbub2*-3, Δ*Fgbfa1*-5 and Δ*Fgbfa1*-8) were used for further phenotypic analyses. Furthermore, *FgBUB2* and *FgBFA1* genes were fused with GFP under the influence of their respective native promoters and transformed into the Δ*Fgbub2*-2 and Δ*Fgbfa*-5 mutants, respectively, to generate the complemented strains Δ*Fgbub2*-C and Δ*Fgbfa*-C.

**Fig 5:**
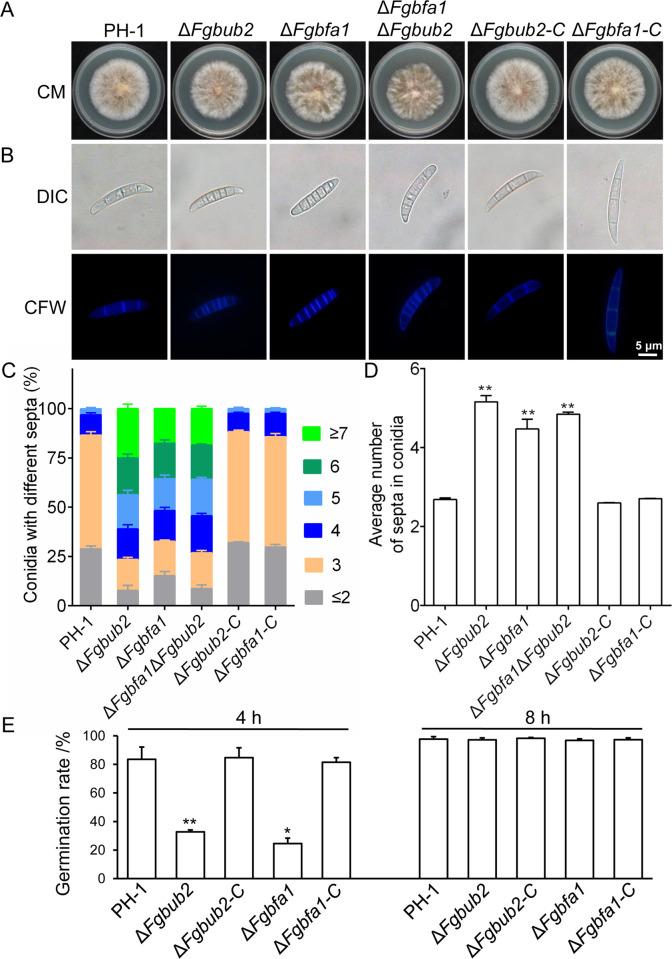
FgBub2 and FgBfa1 negatively regulate conidiation and septum formation. (A) Colony morphologies of the wild type (PH-1), *FgBUB2* deletion mutant (Δ*Fgbub2*), *FgBFA1* deletion mutant (Δ*Fgbfa1*), *FgBUB2-FgBFA1* double deletion mutant (Δ*Fgbfa1*Δ*Fgbub2*) and the complemented strains (Δ*Fgbub2*-C, Δ*Fgbfa1*-C) grown on CM at 28°C for 3 days. (B) Conidial morphology and number of septa in the PH-1, Δ*Fgbub2*, Δ*Fgbfa1*, Δ*Fgbfa1*Δ*Fgbub2*, Δ*Fgbub2*-C and Δ*Fgbfa1*-C strains after incubation in liquid CMC for 3 days and observed under a fluorescence microscope. Conidia were stained with 10 μg ml^-1^ of calcofluor white (CFW). (C) Percentages of conidia with different numbers of septa in the indicated strains. More than 100 conidia from each strain were counted in each experiment. Error bars represent standard deviations from three replicates. (D) The average number of septa in the conidia from each strain. More than 100 conidia of the indicated strains were counted in each experiment. Error bars represent standard deviation from three replicates. Two-tailed Student *t*-test was used for paired comparison of the number of septa from each mutant and the PH-1 (**P < 0.01). (E) Germination of PH-1 and the indicated deletion mutants was measured by determining the percentage of germinated conidia in liquid CM culture after 4 and 8 h of incubation, respectively. (**P < 0.01 or *P < 0.05).

We first examined the colony morphologies and vegetative growths of the Δ*Fgbub2* and Δ*Fgbfa1* mutants on complete media (CM) after 3 days of incubation. Their morphologies were found to be similar to the PH-1, Δ*Fgbub2*-C and Δ*Fgbfa1*-C strains (Fig 5A), despite a slight reduction in the colony diameters of Δ*Fgbub2* and Δ*Fgbfa* mutants (Table 2), suggesting that FgBub2 and Fgbfa are dispensable for the vegetative growth of *F. graminearum*.

**Table 2:** Phenotypic characterization of *FgBUB2* and *FgBFA1* deletion mutants.

Strain	Colony diameter (cm)^1^	Conidiation (10^4^ ml^-1^)^2^	Disease index^3^
*PH-1*	6.26±0.04	205.17±16.41	15.28±1.27
Δ*Fgbub2*	5.92±0.04^**^	56.50±12.70^**^	11.46±2.71^*^
Δ*Fgbfa1*	5.73±0.06^**^	50.83±12.59^**^	11.83±1.64^*^
Δ*Fgbfa1*Δ*Fgbub2*	5.43±0.06^**^	34.33±10.92^**^	11.44±1.58^*^
Δ*Fgbub2-C*	6.28±0.04	206.17±20.83	15.01±1.40
Δ*Fgbfa1-C*	6.22±0.02	208.33±15.56	15.22±1.71

1 Colony diameter of the indicated strains on CM agar plates after 3 days incubation at 28°C.

2 Quantification of the conidial production of the indicated strains in CMC media cultured for 3 days.

3 Disease index was rated by the number of symptomatic spikelet 14 days after inoculation.

Mean and standard error were calculated from three independent experiments. Two-tailed Student’s *t*-test was used for paired comparison between Δ*Fgbub2*, Δ*Fgbfa1* or Δ*Fgbfa1*Δ*Fgbub2* strains vs PH-1 (*P<0.05, **P<0.01).

### FgBub2 and FgBfa1 are negative regulators of conidial septation

The conidia produced by *F. graminearum* serves as the inoculum for initiating infection on flowering wheat heads [39]. Next, we inoculated PH-1, Δ*Fgbub2*, Δ*Fgbfa1*, Δ*Fgbub2*-C and Δ*Fgbfa1*-C strains in carboxymethylcellulose (CMC) media for 3 days (Table 2), after which conidia were harvested from the liquid cultures, respectively. Compared to the PH-1 and the complemented strains, the Δ*Fgbub2* and Δ*Fgbfa1* mutants conidiation was reduced by over 70% (Table 2). Interestingly, we found that the number of septa per conidium increased significantly in the Δ*Fgbub2* and Δ*Fgbfa1* mutants’ conidia (Fig 5B). Calcofluor white (CFW) staining bioassays revealed that more than 50% of the conidia produced by the Δ*Fgbub2* and Δ*Fgbfa1* mutant strains have at least 5 septa (Fig 5C), while less than 10% of the conidia produced by the wild type and the complemented strains have this number of septa per conidium (Fig 5C). Furthermore, over 20% of the conidia produced by the Δ*Fgbub2* and Δ*Fgbfa1* strains have at least 7 septa (Fig 5C) while only very few in the PH-1 and the complemented strains have this property (Fig 5C). Quantitative statistical analyses showed that the average number of septa per conidium in the Δ*Fgbub2* and Δ*Fgbfa1* mutants was more than 4 while only an average of less than 3 was observed in the PH-1 (Fig 5D). Moreover, as shown in Fig 5E, the conidial germination rate of Δ*Fgbub2* and Δ*Fgbfa1* mutants was only approximately 30%, significantly decreased compared with the wild type after 4 h of incubation. When extended to 8 h, more than 95% of the conidia of Δ*Fgbub2* and Δ*Fgbfa1* mutants can germinate (Fig 5E), suggesting a delayed conidial germination in Δ*Fgbub2* and Δ*Fgbfa1* mutants. Taken together, these results indicate that FgBub2 and FgBfa1 negatively regulate the conidium septum formation and thus could be involved in the septation initiation network (SIN) in *F. graminearum*.

### FgBub2 and FgBfa1 play negative roles in regulating nuclear distribution and division

**Fig 6:**
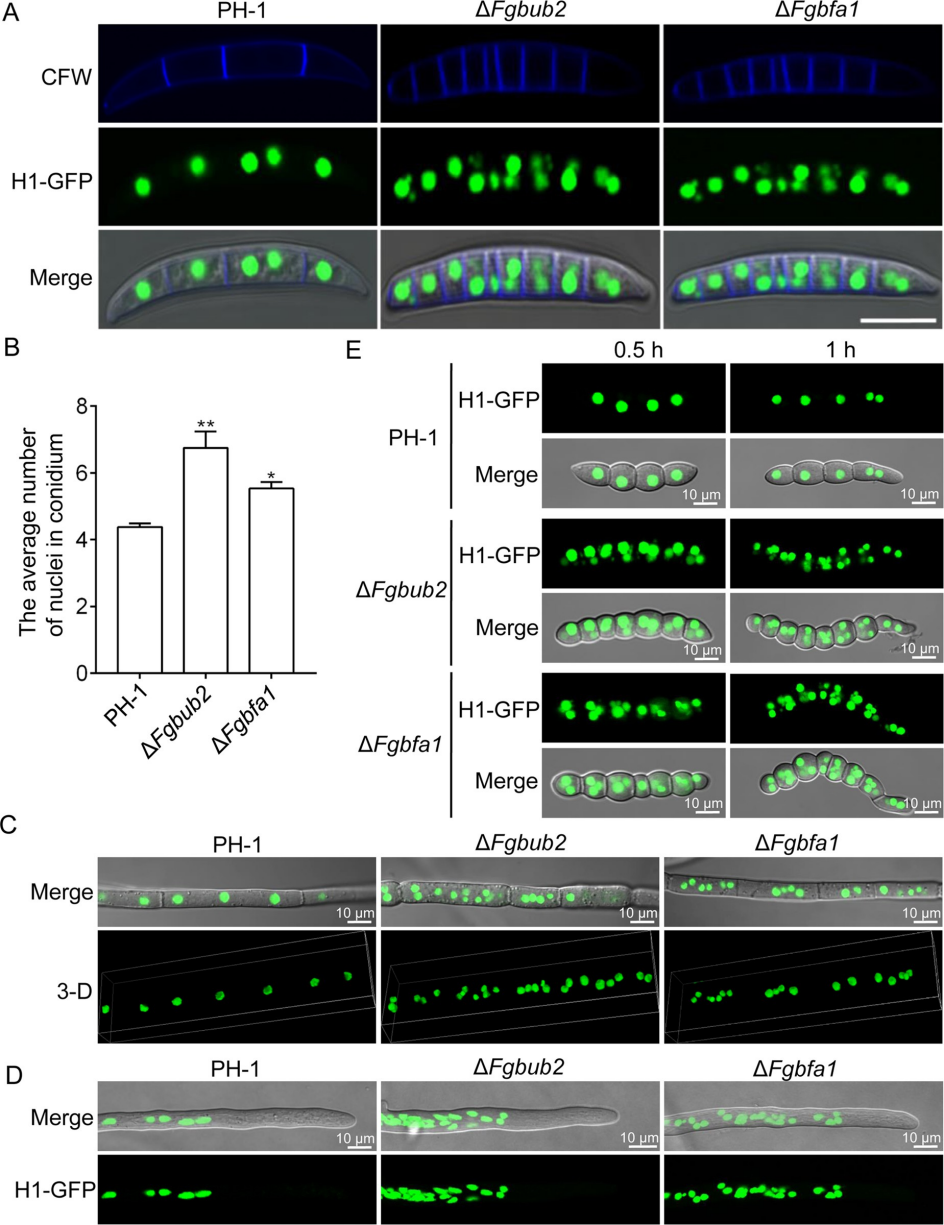
FgBub2 and FgBfa1 are required for normal nuclear distribution and division. (A) Conidia from the PH-1, Δ*Fgbub2* and Δ*Fgbfa1* strains expressing FgHistone1-GFP (H1-GFP) constructs. The images showing the distribution of nuclei in the conidia were taken after 3 days of incubation in CMC media under a fluorescence microscope. The conidia were stained with 10 μg ml^-1^ of calcofluor white (CFW) prior to microscopy. (B) Statistical analysis of the average number of nuclei in conidium from three repeated experiments, at least 100 conidia of the indicated strains were counted in each experiment. Error bars represent the SD. Two-tailed Student *t*-test was used for paired comparison of the number of nuclei from different mutants and PH-1, respectively. (**P < 0.01 or *P < 0.05). (C-D) The distribution of nuclei in the basal and tip hyphae of PH-1, Δ*Fgbub2* and Δ*Fgbfa1* strains. Nuclei were distributed evenly along the hyphae in the wild type, while clumped nuclei were frequently observed in the Δ*Fgbub2* and Δ*Fgbfa1* mutants. (F) The distribution of nuclei in germinating conidia (0.5 and 1 h) of PH-1, Δ*Fgbub2* and Δ*Fgbfa1* strains.

Based on the earlier observations that FgBub2 and FgBfa1 negatively regulate septum formation, we speculated that these two genes are likely important for the progression of cell division in *F. graminearum*. To determine the roles of FgBub2 and FgBfa1 in nuclear division, we transformed H1-GFP into the protoplasts of the Δ*Fgbub2* and Δ*Fgbfa1* mutants, respectively. As shown in Fig 6, the number of nuclei significantly increased in the conidia of Δ*Fgbub2* and Δ*Fgbfa1* mutants in comparison to those in the PH-1 (Fig 6A and 6B), the majority of the conidia from the PH-1 have only one nucleus (rarely two) in each inter-septal compartment, but multiple nuclei were observed in each inter-septal compartment of Δ*Fgbub2* and Δ*Fgbfa1* mutants (Fig 6A). Furthermore, we also examined the distribution of nuclei in the basal and tip hyphae of the Δ*Fgbub2*, Δ*Fgbfa1* and PH-1 strains. As shown in Fig 6C and 6D, the nuclei were distributed evenly along the hyphae in the PH-1, while clumped nuclei were frequently observed in the Δ*Fgbub2* and Δ*Fgbfa1* mutants (S2–S4 Videos). The numbers of nuclei in the Δ*Fgbub2* and Δ*Fgbfa1* mutants are significantly increased in comparison to those in the PH-1. There are averagely 3.7 nuclei between any two successive septa in the PH-1 while an average of 5.11 and 5.01 nuclei were observed between such septa in the Δ*Fgbub2* and Δ*Fgbfa1* mutants, respectively (Fig 6C), suggesting an abnormal nuclear division in the Δ*Fgbub2* and Δ*Fgbfa1* mutants. We further observed the nuclei in germinating conidia of the Δ*Fgbub2*, Δ*Fgbfa1* and PH-1 strains, the result showed that the numbers of nuclei in the Δ*Fgbub2* and Δ*Fgbfa1* mutants are also significantly increased in comparison to those in the PH-1 (Fig 6E). Taken together, we conclude from these results that FgBub2 and FgBfa1 negatively regulate nuclear distribution and division in *F. graminearum*.

### FgBub2 and FgBfa1 play a critical role in sexual reproduction

**Fig 7:**
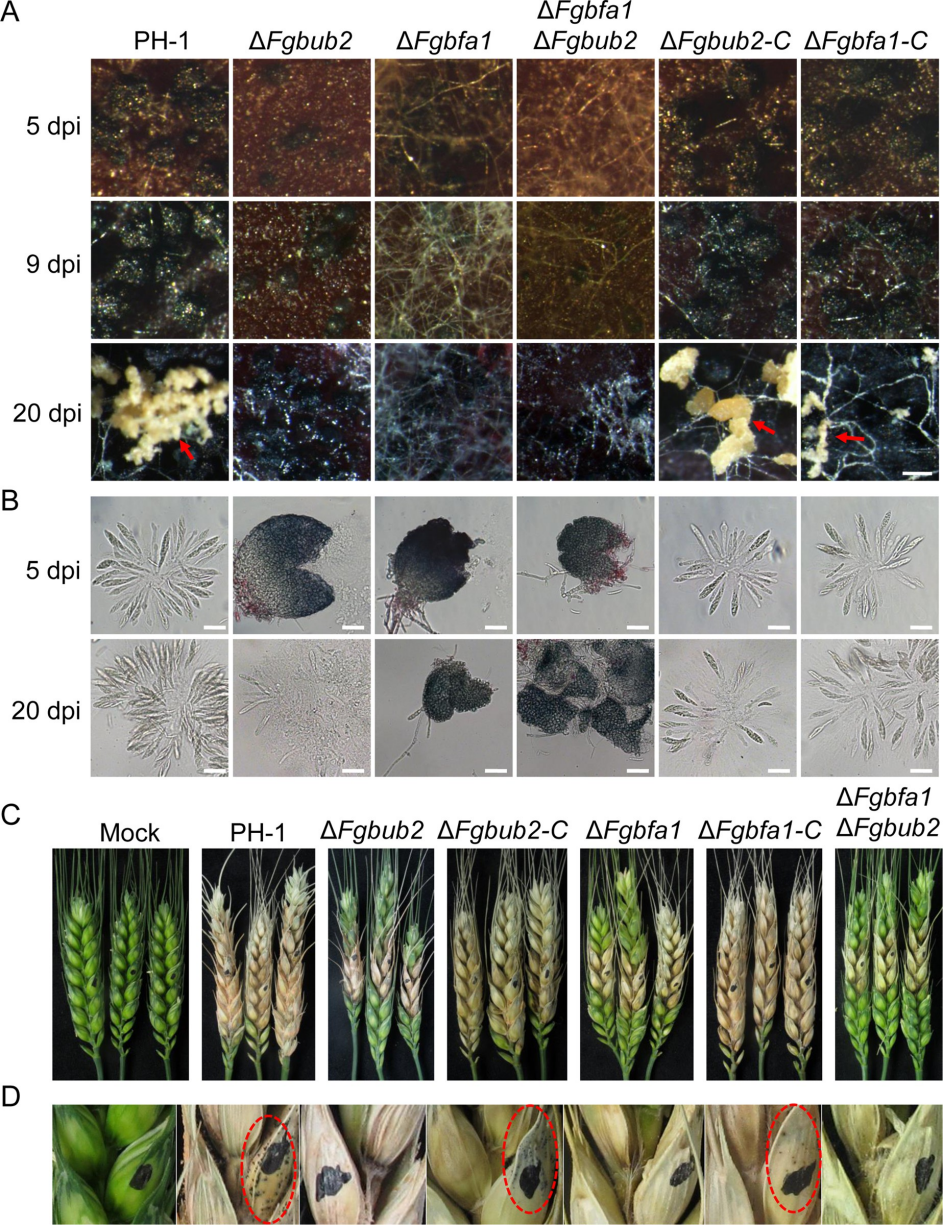
Effects of *FgBUB2* and *FgBFA1* deletions on sexual reproduction and pathogenicity of *F. graminearum*. (A) The PH-1, Δ*Fgbub2*, Δ*Fgbfa1*, Δ*Fgbfa1*Δ*Fgbub2*, Δ*Fgbub2*-C and Δ*Fgbfa1*-C strains were examined for perithecia and cirrhus production on carrot agar medium at 5, 9 and 20 dpi (day post-inoculation). Bar = 200 μm. (B) Asci and ascospores formed by PH-1, Δ*Fgbub2*-C and Δ*Fgbfa1*-C strains. The Δ*Fgbub2*, Δ*Fgbfa1* and Δ*Fgbfa1*Δ*Fgbub2* mutants failed to produce mature ascospores, Bars = 50 μm. Photographs were taken at 5 and 20 dpi. (C) Pathogenicity of the wild type PH-1, indicated mutants and complemented strains on wheat heads. Infected wheat heads were examined at 14 dpi. (D) Perithecia produced by the PH-1, Δ*Fgbub2*-C and Δ*Fgbfa1*-C strains. Perithecia were not found in the wheat heads infected by Δ*Fgbub2*, Δ*Fgbfa1* and Δ*Fgbfa1*Δ*Fgbub2* mutants.

*F. graminearum* is a homothallic fungus and its perithecia and ascospores play critical roles in its disease cycle [40]. To determine the role of FgBub2 and FgBfa1 in sexual reproduction, we inoculated the PH-1, Δ*Fgbub2*, Δ*Fgbfa1* and the complemented strains Δ*Fgbub2*-C and Δ*Fgbfa1*-C on carrot media to induce perithecium and ascospore formations. As shown in Fig 7A and 7B, PH-1, Δ*Fgbub2*-C and Δ*Fgbfa1*-C strains produced abundant mature perithecia and normal ascospores at 5 dpi (days post-inoculation), while the Δ*Fgbub2* and Δ*Fgbfa1* mutants produced very few perithecia and failed to form ascospores. When the incubation period was extended to 9 dpi or 20 dpi, the Δ*Fgbub2* and Δ*Fgbfa1* mutants were similarly observed to produce fewer perithecia in comparison to those in the PH-1, Δ*Fgbub2*-C and Δ*Fgbfa1*-C strains (Fig 7A). Interestingly, there were cirrhi (ascospores oozing) ejecting from the perithecia in the PH-1, Δ*Fgbub2*-C and Δ*Fgbfa1*-C strains, while such cirrhi could not be observed in the Δ*Fgbub2* and Δ*Fgbfa1* mutants (Fig 7A). Also, the Δ*Fgbub2* and Δ*Fgbfa1* mutants were still defective in ascospore formation even at 20 dpi (Fig 7B). Taken together, these results suggest that FgBub2 and FgBfa1 are both required for normal sexual reproduction in *F. graminearum*.

### FgBub2 and FgBfa1 are required for pathogenicity and DON production

To assay for the pathogenicity of the *FgBUB2* and *FgBFA1* gene deletion mutants on flowering wheat heads, we inoculated PH-1, Δ*Fgbub2*, Δ*Fgbfa1*, Δ*Fgbub2*-C and Δ*Fgbfa1*-C strains on flowering wheat heads, respectively, for 14 days. The PH-1 and the complemented strains Δ*Fgbub2*-C and Δ*Fgbfa1*-C caused serious head blight symptoms in the inoculated florets and spread to the entire spikelets, while those inoculated with Δ*Fgbub2* and Δ*Fgbfa1* mutants had low progression of the head blight symptoms spreading from the inoculated florets to nearby spikelets less than the PH-1 (Fig 7C and Table 2). As a positive control, the PH-1 showed an average disease index (diseased spikelets per head) of approximately 15.28, while the Δ*Fgbub2* and Δ*Fgbfa1* mutants had less than 12 (Table 2). Moreover, perithecia were found in the wheat spikelets inoculated with mycelia from the PH-1 and complemented strains Δ*Fgbub2*-C and Δ*Fgbfa1*-C. However, there were no visible perithecia found in the wheat spikelets inoculated with mycelia from the Δ*Fgbub2* and Δ*Fgbfa1* mutants (Fig 7C and 7D). Taken together, these results suggest that FgBub2 and FgBfa1 are both required for effective plant infection and perithecia formation in the field.

**Fig 8:**
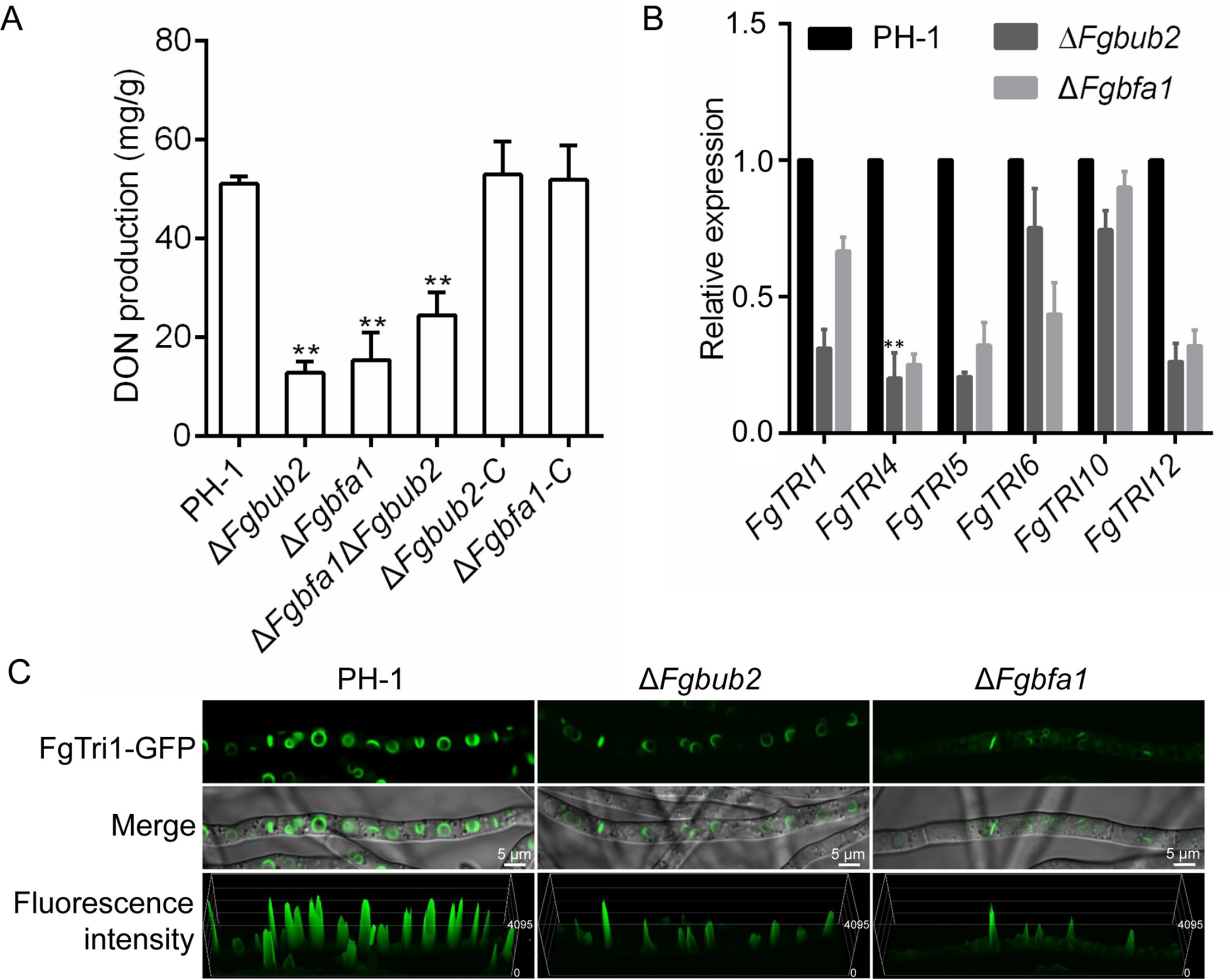
FgBub2 and FgBfa1 are both required for DON production in *F. graminearum*. (A) Amounts of DON produced by the wild type PH-1, Δ*Fgbub2*, Δ*Fgbfa1*, Δ*Fgbfa1*Δ*Fgbub2*, Δ*Fgbub2*-C and Δ*Fgbfa1*-C strains in liquid TBI media after incubation for 7 days. The Δ*Fgbub2*, Δ*Fgbfa1* and Δ*Fgbfa1*Δ*Fgbub2* mutants had significantly reduced levels of DON. Asterisks indicate significant differences. Two-tailed Student *t*-test was used for paired comparison of the DON produced by PH-1 and the different mutants, respectively. (**P < 0.01). (B) The relative expression levels of *FgTRI1*, *FgTRI4*, *FgTRI5*, *FgTRI10* and *FgTRI12* genes in the PH-1, Δ*Fgbub2* and Δ*Fgbfa1* strains cultured in liquid TBI media for 3 days. Two-tailed Student *t*-test was used for paired comparison of the TRI genes in PH-1 and the different mutants, respectively. (**P < 0.01 or *P < 0.05). (C) The expression of FgTri1-GFP in PH-1, Δ*Fgbub2* and Δ*Fgbfa1* strains. FgBub2 and FgBfa1 are dispensable for toxisome formation. However, the fluorescence intensity of the FgTri1-GFP in the Δ*Fgbub2* and Δ*Fgbfa1* mutants are lower than those in the PH-1.

DON is one of the best-characterized virulence factors in *F. graminearum* [9]. To test whether FgBub2 and FgBfa1 are required for DON production, we inoculated the PH-1, Δ*Fgbub2* and Δ*Fgbfa1* mutants in liquid TBI media and incubated at 28°C for 7 days in the dark, after which we assayed for the total DON produced by the strains. We found that Δ*Fgbub2* and Δ*Fgbfa1* mutants showed significantly lower levels of DON production than the PH-1 (Fig 8A), suggesting that FgBub2 and FgBfa1 are both involved in DON production. To gain further insights into the roles of FgBub2 and FgBfa1 in DON biosynthesis, we monitored the expression levels of some DON biosynthesis genes, including *FgTRI1*, *FgTRI4*, *FgTRI5*, *FgTRI6*, *FgTRI10* and *FgTRI12* in the Δ*Fgbub2* and Δ*Fgbfa1* mutant strains, cultured in TBI media, using qRT-PCR. As shown in Fig 8B, the expression levels of these six genes in Δ*Fgbub2* and Δ*Fgbfa1* mutants are decreased compared to those in the PH-1. Recent studies suggested the existence of some subcellular compartments (called toxisomes) that harbor the DON biosynthesis enzymes in *F. graminearum*, and that FgTri1 serves as a marker for the toxisomes [41]. As such, we went further to check whether FgBub2 and FgBfa1 are involved in toxisome biogenesis by transforming FgTri1-GFP into the protoplasts of the PH-1, Δ*Fgbub2* and Δ*Fgbfa1* mutants and then examine the FgTri1-GFP signal in the various strains. As shown in Fig 8C, toxisomes were detected in all the strains but the fluorescence intensity of the FgTri1-GFP signal in the Δ*Fgbub2* and Δ*Fgbfa1* strains appears weaker than that in the PH-1 under the same condition, suggesting that FgBub2 and FgBfa1 are dispensable for the toxisome formation. Taken together, these results indicate that FgBub2 and FgBfa1 play key roles in trichothecene biosynthesis genes expression and DON production in *F. graminearum*.

### Functional characterization of *FgBUB2* and *FgBFA1* double deletion mutant in *F. graminearum*

FgBub2 directly interacts with FgBfa1 to form a heterodimer in *F. graminearum* (Fig 4B). However, it remains unknown whether these two genes have functional redundancy. To test the possibility of functional redundancy of FgBub2 and FgBfa1, we knocked out the *FgBFA1* gene in the Δ*Fgbub2* mutant and obtained *FgBFA1* and *FgBUB2* double deletion mutants (Δ*Fgbfa1*Δ*Fgbub2*) (S2D Fig). Phenotypic characterization of the Δ*Fgbfa1*Δ*Fgbub2* mutants showed that the vegetative growth and conidiation of Δ*Fgbfa1*Δ*Fgbub2* strain were slightly decreased in comparison to those in the *FgBFA1* and *FgBUB2* single-gene deletion strains (Fig 5A and Table 2). Additionally, we found that sexual reproduction, conidial septation, DON production and pathogenicity of the Δ*Fgbfa1*Δ*Fgbub2* strains were very similar to those of the *FgBFA1* and *FgBUB2* single-gene deletion strains (Figs 5B, 5D, 7 and 8A). Taken together, these results indicate a little redundancy in the functions of FgBub2 and FgBfa1 in *F. graminearum*.

### Genetic relationship of FgBub2 and FgBfa1 with FgTem1

**Fig 9:**
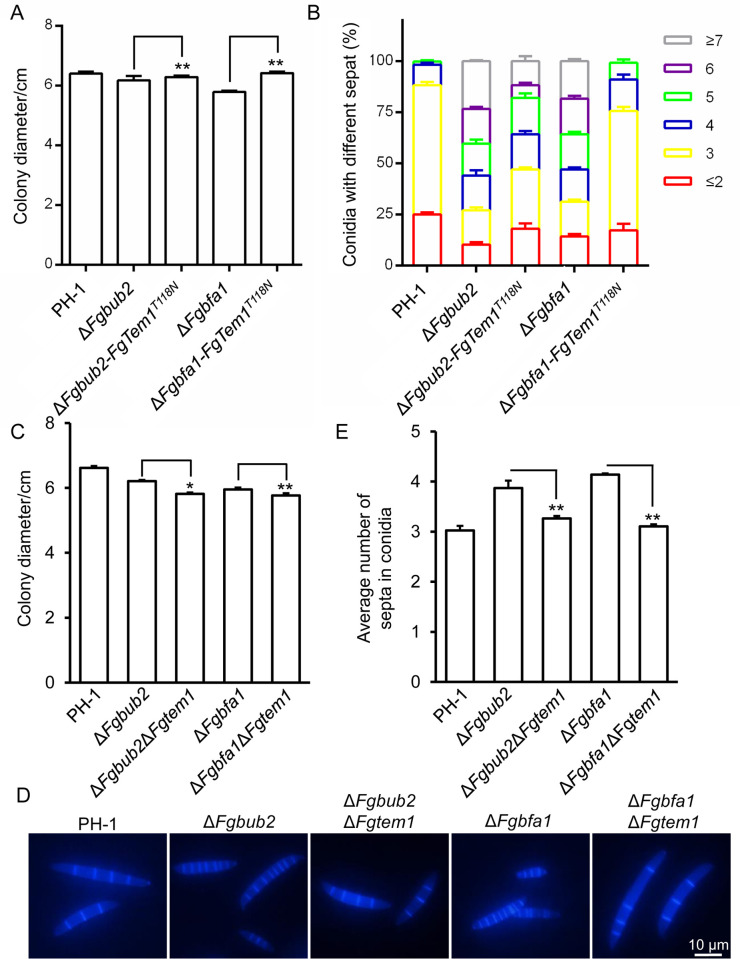
Genetic relationship of FgBub2 and FgBfa1 with FgTem1. (A) The dominant negative isoform of FgTem1 (FgTem1^T118N^) partially rescues the vegetative growth of Δ*Fgbub2* and Δ*Fgbfa1* mutants. Two-tailed Student *t*-test was used for paired comparison of the colony diameters of Δ*Fgbub2* and Δ*Fgbub2*-FgTem1^T118N^, Δ*Fgbfa1* and Δ*Fgbfa1*-FgTem1^T118N^, (**P < 0.01). (B) Expression of FgTem1^T118N^ partially rescues the number of septa in Δ*Fgbub2* and Δ*Fgbfa1* mutant conidia. (C) Colony diameters of the PH-1, Δ*Fgbub2*, *FgBUB2-FgTEM1* double deletion mutant (Δ*Fgbub2*Δ*Fgtem1*), Δ*Fgbfa1*, and *FgBFA1-FgTEM1* double deletion mutant (Δ*Fgbfa1*Δ*Fgtem1*) grown on CM at 28°C for 3 days. (**P < 0.01 or *P < 0.05). (D-E) Conidial morphologies and number of septa in the PH-1, Δ*Fgbub2*, Δ*Fgbub2*Δ*Fgtem1*, Δ*Fgbfa1*, Δ*Fgbfa1*Δ*Fgtem1* strains after incubation in liquid CMC for 3 days and observed under a fluorescence microscope. (**P < 0.01).

The small GTPase Tem1 cycles between GTP-bound and GDP-bound conformations, GAPs mediate the hydrolysis of GTP to GDP to inactivate Tem1 [34,38,42]. Disruption of a Tem1 GAP may bring about a reduction in the level of down-stream GDP-bound Tem1 and result in accumulation of more up-stream GTP-bound Tem1. Thus, over-expression of a GDP-bound dominant negative form Tem1 in the GAP deletion mutant should at least partially compensate/rescue the phenotypic abnormalities associated with GAP deletion. Therefore, to investigate whether the two-component GAP FgBub2/FgBfa1 targets the GTPase FgTem1 to regulate septum formation in *F. graminearum*, we generated a dominant negative isoform of FgTem1 (pFgTem1^T118N^) analogous to the *S. pombe* homolog Spg1 and *C. orbiculare* CoTem1 [31,37]. Then, we transformed the pFgTem1^T118N^ vector into the protoplasts of Δ*Fgbfa1* and Δ*Fgbub2* mutants. The resulting transformants were examined for vegetative growth and septum formation. As shown in Fig 9A and 9B, the colony diameters and the number of septa in the conidia of Δ*Fgbub2-FgTem1*^T118N^ and Δ*Fgbfa1*-*FgTem1*^T118N^ strains were partially rescued in comparison to those in the Δ*Fgbub2* and Δ*Fgbfa1* mutants, respectively.

Furthermore, to determine whether the loss of *FgTEM1* in either *FgBUB2* or *FgBFA1* deletion mutants suppresses the conidial septation phenotype in *F. graminearum*, we knocked out *FgTEM1* gene in *FgBUB2* or *FgBFA1* single deletion mutants, respectively. As shown in Fig 9C, the vegetative growth of Δ*Fgbub2*Δ*Fgtem1* or Δ*Fgbfa1*Δ*Fgtem1* double deletion mutants slightly decreased compared to Δ*Fgbub2* or Δ*Fgbfa1* single-gene deletion mutants, respectively. Furthermore, we found that the number of septa per conidium of Δ*Fgbub2*Δ*Fgtem1* or Δ*Fgbfa1*Δ*Fgtem1* mutants was significantly decreased compared to that in Δ*Fgbub2* or Δ*Fgbfa1* mutants, and almost restored to the wild type level, respectively (Fig 9D and 9E). This result provides complementary functional evidence that the GAPs FgBub2 and FgBfa1 target FgTem1 in *F. graminearum*. Taken together, these results suggest that the Bub2-Bfa1 heterodimer acts as a GAP for FgTem1 in *F. graminearum*.

## Discussion

**Fig 10:**
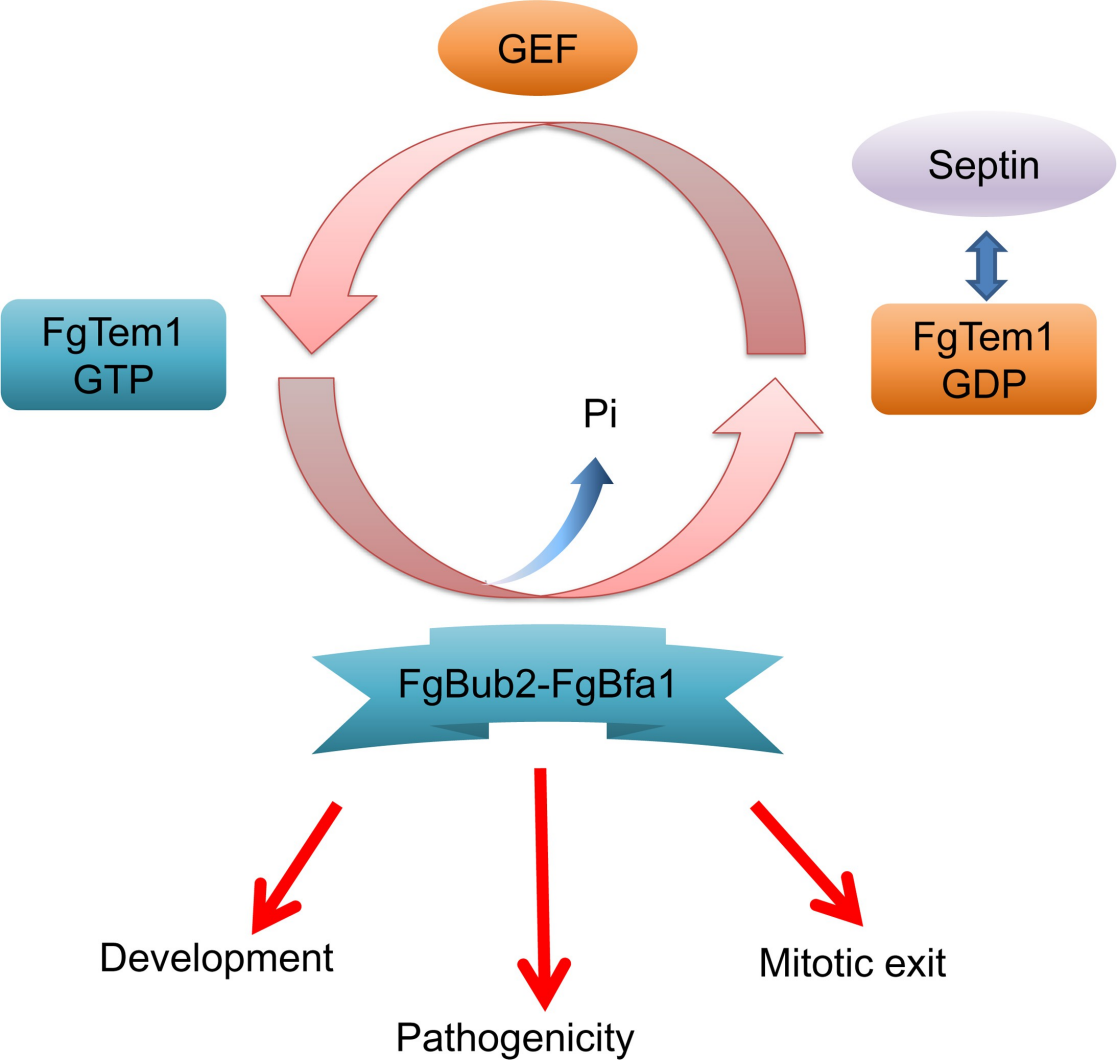
A proposed model of FgBub2-FgBfa1 regulates the small GTPase FgTem1 mediated mitotic exit network and pathogenicity in *Fusarium graminearum*. FgBub2 directly interacts FgBfa1 and acts as a GAP complex of FgTem1 to regulate the development, mitotic exit and pathogenicity in *F. graminearum*. Septin components FgCdc10 and FgCdc11 directly interact with the dominant negative form of FgTem1, FgCdc11 regulates the localization of FgTem1.

The small GTPase Tem1 is involved in mitotic exit network (MEN) and plays a critical role in regulating cell division in yeast [38]. However, the function of Tem1 in phytopathogenic fungi is still largely unknown. In this study, we found that FgTem1 plays a limited role in vegetative growth while the pathogenicity of Δ*Fgtem1* mutant decreased dramatically. Moreover, the regulatory mechanisms of FgTem1 functions have been further investigated (Fig 10). Septin components FgCdc10 and FgCdc11 were demonstrated to interact with the dominant negative form of FgTem1, and FgCdc11 regulates the localization of FgTem1. The cell cycle arrest protein heterodimer FgBub2-FgBfa1 was demonstrated to act as GAP for FgTem1. Septins were shown to be involved in nuclear division, morphogenesis and pathogenicity in *F. graminearum* [17]. The FgBub2-FgBfa1 heterodimer is involved in MEN and the septation initiation network (SIN). Importantly, FgBub2 and FgBfa1 are required for fungal development and pathogenicity. To our knowledge, this work is the most comprehensive genetic dissection of FgTem1 in regulating both physiological developments and pathogenicity in a filamentous fungus.

In the basidiomycete fungus *U. maydis*, a Tem1-like GTPase UmRas3 participates in envelope removal [33], but not required for morphology and cytokinesis. The roles of Tem1 homologs have not been reported in other phytopathogenic fungi. In this study, we found that FgTem1 slightly influence in vegetative growth of *F. graminearum*, but pathogenicity is dramatically reduced in the absence of the protein. Sexual reproduction and DON production were not altered in the Δ*Fgtem1* mutant. Furthermore, according to previous microarray data from *F. graminearum* [43,44], the expression level of *FgTEM1* during wheat head infection is higher than that observed from *in vitro* experiments during sexual development and DON induction conditions (S1 Table). This is consistent with the observed role of FgTem1 in pathogenicity. We further demonstrated that FgTem1 is required for the formation of infection structures and invasive hyphal growth of *F. graminearum* in wheat spikelets and wheat coleoptile cells. Taken together, we conclude that FgTem1 plays important roles in plant infection and pathogenicity of *F. graminearum*.

Septins were first identified in 1970s in budding yeast [45]. Tem1 controls actomyosin and septin dynamics during cytokinesis [46]. Septins have a role of controlling mitotic exit in yeast [47]. Septins were also reported to act as a boundary to restrict to the Tem1 GEF Lte1 [47,48]. However, the relationship between septins and FgTem1 is still unclear. In this study, the septin components FgCdc3, FgCdc10 and FgCdc11 were identified in the FgTem1-GFP pull-down. Furthermore, FgCdc10 and FgCdc11 were demonstrated to specifically interact with the dominant negative GDP-bound form of FgTem1, suggesting that FgCdc10 and FgCdc11 could associate with the dominant negative form of FgTem1 in *F. graminearum*. Furthermore, FgCdc11 regulates the localization of FgTem1. Since septins could act as a mitotic scaffold [49], we speculated that FgCdc10 and FgCdc11 may function as a scaffold for FgTem1 upon which the guanine nucleotide exchange factors provide GEF activity.

Besides septins, FgBub2 was also identified from the FgTem1-GFP pull-down data. Bub2/Bfa1 is a two-component GAP protein of Tem1 in yeast and *C. orbiculare* [27,31]. Yeast two-hybrid assay further showed that FgBfa1 directly interacts with FgBub2 and the constitutively active form of FgTem1. Together with the results showing that FgTem1^T118N^ partially complements the defects of Δ*Fgbub2* and Δ*Fgbfa1* mutants in vegetative growth and septum formation, deletion of *FgTEM1* in either Δ*Fgbub2* or Δ*Fgbfa1* deletion mutants suppresses the conidial septation phenotype, provides complementary functional evidence that the GAPs FgBub2 and FgBfa1 target FgTem1. We conclude that FgBub2-FgBfa1 heterodimer functions as a potential GAP for FgTem1 in *F. graminearum*. Furthermore, double deletion mutants of *FgBUB2* and *FgBFA1* genes were observed to exhibit similar phenotypes to the respective single gene deletion mutants, supporting the hypothesis that the FgBub2 and FgBfa1 proteins form a heterodimer or a two-component GAP.

In filamentous fungi, septum formation and nuclear division are important features of cell division [21]. Proper regulation of cell cycle progression is important for development and pathogenesis of fungal pathogens [50]. There are emerging evidences demonstrating the important roles of Bub2 during plant-microbe interactions. In *M. oryzae* and *C. higginsianum*, *BUB2* regulates G1/S transition and septum formation, and the regulation of these processes are required for successful plant infection [32,51]. In *C. orbiculare*, CoBub2 interacts with CoBfa1 and is required for appressorium-mediated plant invasion [31]. However, *Cobub2*Δ and *Cobfa1*Δ mutants have no defective septation [31]. Herein, we found that deletion of *FgBUB2* caused a slightly delayed growth but significantly attenuates the pathogenicity of *F. graminearum*, similar to the pathogenicity defects reported for *CoBUB2* deletion mutants generated from *C. orbiculare* [31]. Moreover, disruption of *FgBUB2* or *FgBFA1* gene results in increased number of septa and nuclei, and these were not observed in *C. orbiculare* [31], suggesting the diverse functions of Bub2 and Bfa1 in different phytopathogenic fungi. We further demonstrated that disruption of nuclear division arrest delayed conidial germination. In addition, deletion of *FgTEM1* has such a small effect on nuclear division and septation compared with *FgBUB2* or *FgBFA1* gene deletion mutants, maybe due to another functionally redundant GTPase in the genome of *F. graminearum* that could overlap with FgTem1 in regulating nuclear division and septation.

Sexual reproduction enables exchange of genetic materials in eukaryotic organisms such as fungi, animals, plants, and ciliates [52]. Sexual reproduction drives genetic recombination throughout eukaryotic organisms and serves to purge deleterious mutations, producing better-adapted progeny [52]. In *F. graminearum*, a perithecium produced by sexual reproduction is important for overwintering in its life cycle, and sexual ascospores released from the perithecium can infect cereal heads during flowering [5,40]. In this study, we noticed that perithecium formation is dramatically affected in the *FgBUB2* and *FgBFA1* deletion mutants on carrot agar media. Cytokinesis is the last step of a cell cycle resulting in generation of two progeny, failure of correct cell division may be lethal for both mother and daughter cells [21]. Asci and ascospores were not found in the perithecia of these mutants, maybe due to abnormal meiosis or cytokinesis in the mutants. Consistently, perithecia were not found in the wheat heads of these mutants in the field, suggesting that *FgBUB2* and *FgBFA1* can serve as potential targets for development of anti-fungal drugs against *F. graminearum* since these processes are critical for the survival and overwinter of the fungus.

Bub2 and Bfa1 localize at the Spindle Pole Bodies (SPB) in yeast [27,53]. However, in this study, the fluorescence signals of FgBub2-GFP and FgBfa1-GFP (expressed under their respective native promoters) are both weak and diffuse in the cytoplasm of the hyphae and conidia (S9 Fig). Furthermore, a GFP sequence was fused to the N-terminus of *FgBUB2* and *FgBFA1* using ToxA promoter which effectively expressed in *F. graminearum* was tested [12,54,55]. Consistently, we found that the GFP signal also diffuse in the cytoplasm (S9 Fig), these results together suggesting a nonspecific localization of FgBub2 and FgBfa1 in *F. graminearum*. In contrast, FgTem1-GFP clearly localizes to SPB in the hyphae, conidiophores and conidia, consistent with Tem1 localization to the spindle pole bodies (SPB) in yeast [35], indicating a conserved localization of FgTem1.

In summary, this study expands our understanding of the roles Tem1 GTPase as well as its regulators in the mitotic exit network in filamentous fungi (Fig 10). We systemically identified the potential regulators of FgTem1 in *F. graminearum* and demonstrated for the first time that FgTem1 plays an important role in the pathogenicity of phytopathogenic fungi. We found that the septin FgCdc11 regulates the localization of FgTem1, and identified the function of the heterodimer FgBub2-FgBfa1 as a GAP for FgTem1 in *F. graminearum*.

## Material and methods

### Strains and culture conditions

The wild type (PH-1), mutants and complemented strains used in this study are all listed in S2 Table. PH-1 and all other strains were grown and evaluated by culturing the strains on complete medium (CM) or starch yeast medium (SYM) at 28°C for 3 days [56,57]. Sexual reproduction was assayed on carrot agar medium according to a previously report [58]. Conidiation was measured in liquid carboxymethylcellulose (CMC) medium as previously reported [59].

### Deletion of *FgTEM1*, *FgBUB2* and *FgBFA1* genes

*F. graminearum* protoplast preparation and fungal transformation were performed following standard protocols [60]. The split-marker approach [61] was used to generate gene replacement constructs for the *FgTEM1*, *FgBUB2* and *FgBFA1* genes. The primers used to amplify the flanking sequences for each gene are listed in S3 Table, knockout candidates were screened by PCR using the primers listed in S3 Table and further verified by Southern blot using Digoxigenin High Prime DNA Labeling and Detection Starter Kit I (Roche). *FgBFA1* and *FgBUB2* double deletion mutants were constructed by deleting *FgBFA1* in the Δ*Fgbub2* mutant using neomycin-resistant marker (G418) and putative Δ*Fgbfa1*Δ*Fgbub2* double deletion mutants were screened by PCR using the primers listed in S3 Table and further verified by Southern blot analysis. The same method was used to obtain the Δ*Fgbub2*Δ*Fgtem1* and Δ*Fgbfa1*Δ*Fgtem1* double gene deletion mutants.

### Construction of pFgTem1-GFP, pFgBub2-Flag, pFgAlp6-mCherry, pFgBub2-GFP, pFgBfa1-GFP, pFgCdc10-GFP and pFgCdc11-GFP fusion vectors and complementation

The pFgTem1-GFP fusion vector, was constructed by amplifying the native promoter of FgTem1 together with its coding sequence using the primer pair FgTem1CF/FgTem1GR (S3 Table), and the PCR product was cloned into pKNTG2 vector using One Step Cloning Kit (Vazyme Biotech Co., Ltd) and verified by sequencing. The vector was finally transformed into the Δ*Fgtem1* mutant for complementation. Transformants were screened by PCR using the primer pairs listed in S3 Table and further confirmed by GFP signal detection analysis. The same method was used to construct pFgBfa1-GFP, pFgBub2-GFP, pFgCdc10-GFP and pFgCdc11-GFP vectors. pFgAlp6 was cloned into pKNT-mCherry using its primer pairs (S3 Table) and verified by sequencing. For pFgBub2-Flag vector, the primers FgBub2-ZF-pCX62 and FgBub2-OR-pCX62 were used to amplify the sequence of *FgBUB2* and then cloned into pCX62 vector and verified by sequencing.

### Construction of pToxA-GFP-FgBub2 and pToxA-GFP-FgBfa1 fusion vectors

The pToxA-GFP-FgBub2 fusion vector was constructed by amplification of FgBub2 coding sequence and its 3’UTR using the primer pairs listed in S3 Table. ToxA-WF-XhoI and GFPR-TAA primer pair was used to amplify the ToxA-GFP fragment from the pCT74 plasmid [54], and the PCR products were cloned into pKNT vector using One Step Cloning Kit and verified by sequence analysis. The same method was adopted for the construction of pToxA-GFP-FgBfa1 vector.

### Yeast two-hybrid assay

pFgBfa1-AD, pFgTem1WT-AD (wild type), pFgTem1CA-AD (Q163L) and pFgTem1DN-AD (T118N) were constructed and cloned into the pGADT7 (prey construct) vector using the primers listed in S3 Table and amplified from the cDNA of PH-1, respectively. pFgBfa1-BD and pFgBub2-BD were amplified from the cDNA of PH-1, respectively, using their respective primer pairs (S3 Table) and cloned into the pGBKT7 (bait construct) vector. pFgCdc3BD, pFgCdc10BD and pFgCdc11BD vectors were reported previously [17]. Yeast two-hybrid assay was carried out according to MATCHMAKER GAL4 Two-Hybrid System 3 (Clontech). Different combinations of yeast two-hybrid plasmids were co-transformed into the yeast strain AH109 with carrier DNA following a previously reported protocol [13]. The yeast transformants were assayed for growth on SD-Trp-Leu-His-Ade medium with X-α-Gal.

### Affinity capture-mass spectrometry analysis, co-immunoprecipitation (Co-IP) assays and western blot

Briefly, conidia from FgTem1-GFP and PH-1-GFP strains were harvested and then suspended in 250 ml liquid CM at a concentration of 10x10^4^ ml^-1^ conidial suspension and incubated at 28°C, 180 rpm for 16 h, respectively. Next, affinity capture-mass spectrometry analysis was followed as previously reported [56]. For Co-IP assay, total proteins of FgBub2-Flag/FgTem1-GFP, FgBub2-Flag/pCT74-sGFP strains were isolated and then incubated with 30 μl of GFP-Trap_A beads according to the manufacturer’s instructions, respectively. Proteins eluted from the GFP-Trap_A beads were analyzed by western blot using an anti-Flag antibody (Abmart, China) and anti-GFP antibody (Abmart, China) as previously reported [55].

### GTPase activity assay

pHis-FgBub2 and pGST-FgTem1 vectors were constructed by amplifying *FgBUB2* or *FgTEM1* coding sequence from the cDNA of PH-1 using the primer pairs listed in S3 Table, then cloned into the pET32a (His) or pGEX-4T (GST) vector, respectively. His-FgBub2, GST-FgTem1 and His proteins were expressed in BL21 *Escherichia coli* strain and purified, respectively. Next, these proteins were used for GAP activity assay using a GTPase assay kit (Sigma-Aldrich, Catalog Number MAK113) according to the manufacturer’s protocols.

### Plant infection and DON production assays

Infection assays on flowering wheat heads were conducted as previously described [12], and symptoms were observed 14 days after inoculation. To observe the invasive hyphae, the indicated strains were grown in the wheat coleoptiles for 1–3 days. Corn silk infection assay was performed as previously reported [62]. To observe the infection structure, infected lemmas sampled at 2 days were fixed and coated with gold–palladium before examination with a scanning electron microscope (SEM), as previously described [63,64]. For DON production assays, all the strains were grown in liquid trichothecene biosynthesis induction (TBI) media at 28°C for 7 days in the dark, after which the liquid and mycelia were collected, respectively. The liquid was used for enzyme linked immunosorbent assay (ELASE), while the mycelia were dried and used to quantify the fungal biomass [65].

### RNA extraction and Quantitative RT–PCR

For RNA extraction, the PH-1, Δ*Fgbub2* and Δ*Fgbfa1* strains were inoculated in liquid TBI media and incubated for 3 days at 28°C in the dark. Extraction of total RNA and subsequent synthesis of first-strand cDNA were performed as previously described [12]. The primer pairs used to amplify the selected genes in qRT-PCR reactions are listed in S3 Table. All qRT-PCR assays were conducted in technical triplicates for each sample, and the experiment was repeated three times.

## Supporting information

S1 Table Expression profiles of *FgTEM1* during sexual development, conidium germination, DON induction and wheat head blight. (DOCX)

S2 Table Wild type (PH-1) and mutant strains of the fungi used in this study. (DOCX)

S3 Table PCR primers used in this study. (DOCX)

S1 Fig Phylogenetic analysis of Tem1 protein in different species. The phylogenetic analysis of Tem1 in *F. graminearum* is shown in relation to other fungal species. The sequence alignments were performed using the Clustal X 1.83 program and the phylogenetic tree was generated based on neighbor-joining method using MEGA 6.0 software with 10000 bootstrap replicates between Tem1 homologues in different organisms. Accession numbers: *Aspergillus flavus* (AfTem1-XP_002376572.1, AfBub2-RAQ49602.1, AfBfa1-XP_002382919.1), *Botrytis cinerea* (BcTem1-EMR82353.1, BcBub2-XP_001559021.1, BcBfa1-EMR88000.1), *Candida albicans* (CaTem1-XP_019330622.1, CaBub2-XP_715368.2, CaBfa1-RLP61346.1), *Colletotrichum orbiculare* (CoTem1-TDZ19252.1, CoBub2-TDZ14987.1, CoBfa1-TDZ23356.1), *Fusarium graminearum* (FgTem1-EYB30459.1, FgBub2-XP_011316822.1, FgBfa1-XP_011324890.1), Fusarium oxysporum (FoTem1-RKK77852.1, FoBub2-SCO79815.1, FoBfa1-RKK73093.1), *Fusarium verticillioides* (FvTem1-XP_018752224.1, FvBub2-XP_018743852.1, FvBfa1-RBR02439.1), *Neurospora crassa* (NcTem1-XP_960671.3, NcBub2-XP_965337.1, NcBfa1-KHE87150.1), *Magnaporthe oryzae* (MoTem1-XP_018752224.1, MoBub2-XP_003710862.1, MoBfa1-XP_003716829.1), *Saccharomyces cerevisiae* (ScTem1-QHB10674.1, ScBub2-ONH76596.1, ScBfa1-AJR73492.1), *Ustilago maydis* (UmBub2-XP_011386392.1, UmBfa1-XP_011389318.1), *Schizosaccharomyces pombe* (SpSpg1-NP_593285.1, SpBub2-NP_593901.1, SpBfa1-NP_593149.1), *Arabidopsis thaliana* (AtTem1-OAP14749.1).
(TIF)

S2 Fig Southern blot analyses of targeted gene deletion mutants. (A) The scheme for split-marker approach based on the targeted gene replacement of *FgTEM1* with hygromycin resistance (*HPH*) gene. Genomic DNAs were extracted from PH-1 and the putative transformants. *Hind* III-digested DNAs showed a 2.12 kb band in the PH-1 and a 2.48 kb band in the mutants. (B) The scheme for split-marker approach based on the targeted gene replacement of *FgBUB2* with *HPH* gene. Genomic DNAs were extracted from PH-1 and the putative transformants. *BgI* II-digested DNAs showed a 1.80 kb band in the PH-1 and a 5.60 kb band in the mutants. (C) The scheme for split-marker approach based on the targeted gene replacement of *FgBFA1* with *HPH* gene. Genomic DNAs were extracted from PH-1 and the putative transformants. *Nco* I-digested DNAs showed a 3.44 kb band in the PH-1 and a 4.04 kb band in the mutants. (D) The scheme for split-marker approach based on the targeted gene replacement of *FgBFA1* with *NEO* gene. Genomic DNAs were extracted from PH-1 and the putative transformants. *Hind* III-digested DNAs showed a 1.69 kb band in the PH-1 and a 5.61 kb band in the mutants.
(TIF)

S3 Fig Corn silk infection assay. A small block of SYM agar cultures of wild type PH-1, Δ*Fgtem1* and Δ*Fgtem1*-C strains were placed on freshly corn silks for 7 days. Δ*Fgtem1* mutant significantly reduced the pathogenicity.
(TIF)

S4 Fig DON production of PH-1, Δ*Fgtem1* and Δ*Fgtem1*-C strains. Two-tailed Student *t*-test was used for paired comparison of the DON produced by Δ*Fgtem1* and PH-1. (P = 0.16, no significant difference).
(TIF)

S5 Fig FgTem1 is not required for sexual reproduction. (A) Perithecia formation of the wild type PH-1, Δ*Fgtem1* and Δ*Fgtem1*-C strains on carrot agar plates after 9 days. (B) The ascospore released from the perithecia of the indicated strains on carrot agar plates after 9 days.
(TIF)

S6 Fig FgTem1-GFP localizes to the periphery of nuclei. (A) The distribution of nuclei (H1-GFP) in the hyphae of the PH-1 and Δ*Fgtem1*. (B) FgTem1-GFP localizes to the periphery of nuclei in different focal planes of conidiophore. (C) 3-D (three-dimensional) micrograph showing FgTem1-GFP close to nuclear periphery.
(TIF)

S7 Fig The colony morphologies and diameters of constitutive activate and dominant negative isoforms of FgTem1 strains. (A-B) Colony morphologies and diameters of the wild type (PH-1), constitutive activate (FgTem1^Q163L^) and dominant negative (FgTem1^T118N^) isoforms of FgTem1 strains grown on CM at 28°C for 3 days.
(TIF)

S8 Fig Phylogenetic analyses of Bub2 and Bfa1 proteins in different fungal species. (A-B) Phylogenetic analyses of FgBub2 and FgBfa1 with other fungal species. Sequence alignments were performed using Clustal X 1.83 program and the phylogenetic tree was generated based on neighbor-joining method using MEGA 6.0 software with 10000 bootstrap replicates between Bub2 and Bfa1 homologues in different organisms.
(TIF)

S9 Fig The subcellular localization of FgBub2 and FgBfa1. (A) FgBub2-GFP is diffuse in the cytoplasm under the native and ToxA promoters. (B) FgBfa1-GFP is diffuse in the cytoplasm under the native and ToxA promoters.
(TIF)

S1 Video Mobility of FgTem1-GFP. (AVI)

S2 Video Mobility of H1-GFP fusion protein in the hyphal tip of wild type PH-1. (AVI)

S3 Video Mobility of H1-GFP fusion protein in the hyphal tip of the Δ*Fgbub2*. (AVI)

S4 Video Mobility of H1-GFP fusion protein in the hyphal tip of the Δ*Fgbfa1*. (AVI)
