# Supplementary material for: The mitotic exit mediated by small GTPase Tem1 is essential for the pathogenicity of Fusarium graminearum
Source: PLoS Pathog. 2023 Mar 16;19(3):e1011255. doi: 10.1371/journal.ppat.1011255 (PMC10047555; doi:10.1371/journal.ppat.1011255)
Supplement: S1 Table — (DOCX) [file ppat.1011255.s001.docx]

**S1 Table. Expression profiles of *FgTEM1* during sexual development, conidium germination, DON induction and wheat head blight.**

| **Gene** | **FG2:** **Carbon and Nitrogen Starvation** | | **FG5: In vitro sexual development** | | | | | **FG7: Conidium germination** | | | **FG14:** **DON induction** | **FG15:** **Gene expression** **during wheat head blight** | | | | | |
| --- | --- | --- | --- | --- | --- | --- | --- | --- | --- | --- | --- | --- | --- | --- | --- | --- | --- |
|  | Carbon | Nitrogen | 24h | 48h | 72h | 96h | 144h | 2h | 8h | 24h | agmatine | 24h | 48h | 72h | 96h | 144h | 192h |
| Fg17139 | 1.08 | 1.05 | 1.05 | 1.01 | 1.03 | 0.99c | 1.00 | 0.87 | 1.01 | 1.00 | 1.01 | 1.17 | 1.75 | 1.82 | 2.20 | 2.15 | 1.83 |
