# Supplementary material for: The mitotic exit mediated by small GTPase Tem1 is essential for the pathogenicity of Fusarium graminearum
Source: PLoS Pathog. 2023 Mar 16;19(3):e1011255. doi: 10.1371/journal.ppat.1011255 (PMC10047555; doi:10.1371/journal.ppat.1011255)
Supplement: S2 Table — (DOCX) [file ppat.1011255.s002.docx]

**S2 Table. Wild type (PH-1) and mutant strains of the fungi used in this study**

| Strain | Genotype description | Reference |
| --- | --- | --- |
| PH-1  Δ*Fgbub2*  Δ*Fgbub2-C*  Δ*Fgbfa1*  Δ*Fgbfa1-C*  Δ*Fgbfa1*Δ*Fgbub2*  PH-1+Histone1-GFP  Δ*Fgbub2*+Histone1-GFP  Δ*Fgbfa1*+Histone1-GFP  Δ*Fgtem1*  Δ*Fgtem1-C* | Wild-type  FGSG_01065 deletion mutant in PH-1  Δ*Fgbub2* strain expressing the pFgBub2-GFP construct  FGSG_06242 deletion mutant in PH-1  Δ*Fgfa1* strain expressing the pFgBfa1-GFP construct  FGSG_06242 deletion mutant in Δ*Fgbub2*  PH-1 strain expressing the pHistone1-GFP construct  Δ*Fgbub2* strain expressing the pHistone1-GFP construct  Δ*Fgbfa1* strain expressing the pHistone1-GFP construct  FGSG_17139 deletion mutant in PH-1  Δ*Fgtem1* strain expressing the pFgTem1-GFP construct | [[1](#_ENREF_1)]  This study  This study  This study  This study  This study  This study  This study  This study  This study  This study |
| Δ*Fgtem1*Δ*Fgbub2* | FGSG_17139 deletion mutant in Δ*Fgbub2* | This study |
| Δ*Fgtem1*Δ*Fgbfa1* | FGSG_17139 deletion mutant in Δ*Fgbfa1* | This study |
| PH-1+FgCdc10-GFP | PH-1 strain expressing the pFgCdc10-GFP construct | This study |
| PH-1+FgCdc11-GFP | PH-1 strain expressing the pFgCdc11-GFP construct | This study |
| Δ*Fgtem1+*FgCdc10-GFP | Δ*Fgtem1* strain expressing the pFgCdc10-GFP construct | This study |
| Δ*Fgtem1+*FgCdc11-GFP | Δ*Fgtem1* strain expressing the pFgCdc11-GFP construct | This study |
| Δ*Fgcdc10+*FgTem1-GFP | Δ*Fgcdc10* strain expressing the pFgTem1-GFP construct | This study |
| Δ*Fgcdc11+*FgTem1-GFP | Δ*Fgcdc11* strain expressing the pFgTem1-GFP construct | This study |
| Δ*Fgtem1*+Histone1-GFP | Δ*Fgtem1* strain expressing the pHistone1-GFP construct | This study |
| Δ*Fgtem1+*FgTem1-GFP  +FgApl6-mCherry | Δ*Fgtem1* strain expressing the pFgTem1-GFP and pFgApl6-mCherry constructs | This study |
| PH-1*+*FgTem1-GFP  *+*FgBub2-Flag | PH-1 strain expressing the pFgTem1-GFP and pFgBub2-Flag constructs | This study |
| PH-1*+* GFP | PH-1 strain expressing the pCT74-sGFP construct | [[2](#_ENREF_2)] |
| Δ*Fgtem1+*GFP | Δ*Fgtem1* strain expressing the pCT74-sGFP construct | This study |
| PH-1*+*pCT74-sGFP  +FgBub2-Flag | PH-1 strain expressing the pCT74-sGFP and pFgBub2-Flag constructs | This study |
| PH-1+FgTri1-GFP | PH-1 strain expressing the pFgTri1-GFP construct | [[3](#_ENREF_3)] |
| Δ*Fgbub2*+FgTri1-GFP | Δ*Fgbub2* strain expressing the pFgTri1-GFP construct | This study |
| Δ*Fgbfa1*+FgTri1-GFP | Δ*Fgbfa1* strain expressing the pFgTri1-GFP construct | This study |
| PH-1+ToxA-GFP-FgBub2 | PH-1 strain expressing the pToxA-GFP-FgBub2 construct | This study |
| PH-1+ToxA-GFP-FgBfa1 | PH-1 strain expressing the pToxA-GFP-FgBfa1 construct | This study |
| Δ*Fgbub2*+FgTem1^T118N^ | Δ*Fgbub2* strain expressing the pFgTem1^T118N^ construct | This study |
| Δ*Fgbfa1*+ FgTem1^T118N^ | Δ*Fgbfa1* strain expressing the pFgTem1^T118N^ construct | This study |

1. Cuomo CA, Gueldener U, Xu JR, Trail F, Turgeon BG, et al. (2007) The Fusarium graminearum genome reveals a link between localized polymorphism and pathogen specialization. Science 317: 1400-1402.

2. Zheng H, Li L, Yu Z, Yuan Y, Zheng Q, et al. (2021) FgSpa2 recruits FgMsb3, a Rab8 GAP, to the polarisome to regulate polarized trafficking, growth and pathogenicity in Fusarium graminearum. New Phytol 229: 1665-1683.

3. Zheng Q, Yu Z, Yuan Y, Sun D, Abubakar YS, et al. (2021) The GTPase-Activating Protein FgGyp1 Is Important for Vegetative Growth, Conidiation, and Virulence and Negatively Regulates DON Biosynthesis in Fusarium graminearum. Front Microbiol 12: 621519.
