# Supplementary material for: The mitotic exit mediated by small GTPase Tem1 is essential for the pathogenicity of Fusarium graminearum
Source: PLoS Pathog. 2023 Mar 16;19(3):e1011255. doi: 10.1371/journal.ppat.1011255 (PMC10047555; doi:10.1371/journal.ppat.1011255)
Supplement: S3 Table — (DOCX) [file ppat.1011255.s003.docx]

**S3 Table. PCR primers used in this study**

| Primer | Sequence（5΄→3΄） | Application |
| --- | --- | --- |
| FGSG_01065 AF  FGSG_01065 AR  FGSG_01065 BF  FGSG_01065 BR  FGSG_01065 OF  FGSG_01065 OR  FGSG_01065 CF  FGSG_01065 GR  FGSG_01065 UA  FGSG_06242 AF  FGSG_06242 AR  FGSG_06242 BF  FGSG_06242 BR  FGSG_06242 OF  FGSG_06242 OR  FGSG_06242 CF  FGSG_06242 GR  FGSG_06242 UA  FGSG_06242 DAR  FGSG_06242 DBF  FGSG_17139 AF  FGSG_17139 AR  FGSG_17139 BF  FGSG_17139 BR  FGSG_17139 OF  FGSG_17139 OR  FGSG_17139 CF  FGSG_17139 GR  FGSG_17139 UA  H853  YG/F  HY/R  HYG/F  HYG/R  FgBub2 ADF  FgBub2 ADR  FgBub2 BDF  FgBub2 BDR  FgBfa1 ADF  FgBfa1 ADR  FgBfa1 BDF  FgBfa1 BDR  FgTem1ADF  FgTem1ADR  FgTem1 BDF  FgTem1 BDR  FgTem1 CA1R  FgTem1 CA2F  FgTem1 DN1R  FgTem1 DN2F | GATGGGAAACTATTTGAAGG  TTGACCTCCACTAGCTCCAGCCAAGCC-  -TGGTATTATTACTAGGGCTGAT  GAATAGAGTAGATGCCGACCGCGGGTT-  -GCGAATCGGAAGTTTATCG  TCTCCAAGTCTATGCTGGTCTA  CGCCCTTCGTCGTCTTCAA  GCTGCCAGGACATTCATACC  AGGGAACAAAAGCTGGGTACC-  -CAACAACGGTGGAAAGGA  GCCCTTGCTCACCATAAGCTT-  -CATTGCGTGAGACACAATCT  AGTTGGTGCTTTGTTCGGTCTG  CAAGCCAACACCCAGATA  TTGACCTCCACTAGCTCCAGCCAAGCC-  -AATTGCGTACAGTCATTCTT  GAATAGAGTAGATGCCGACCGCGGGTT-  -CAGGTGGTTTGGGTTGTT  TTCTCGCTGCCATTGACT  ACGCCTACCACAACTTCA  TGATTCTTTGCCACTGTTC  AGGGAACAAAAGCTGGGTACC-   -CAAGGCTCCTCTCTTTTCTA  GCCCTTGCTCACCATAAGCTT-  -CATCGCCAAATCGGCGAATT  TGTTTAAGTTGGGTTGAATG  AATGGAAATTGTAAGCGTTAATCTAGA-  -AATTGCGTACAGTCATTCTT  TATCGCCTTCTTGACGAGTTCTTCTGA-  -CAGGTGGTTTGGGTTGTT  CATTTCACGCATAAGCAG  TTGACCTCCACTAGCTCCAGCCAAGCC-  -GGTTGAGTCGTCCAGTTT  GAATAGAGTAGATGCCGACCGCGGGTT-  -GACACGGCCATTTACGAG  TGCTCCGCTGTCTACCTT  AGGCATACAACGACTACCG  GAACTGGCATACCTGATTT  AGGGAACAAAAGCTGGGTACC-  -GAAGCTATTAAGACGCACGA  GCCCTTGCTCACCATAAGCTT-  -ACAGACTGATATAACAGGA  GACAACACGCAAACGAAC  GACAGACGTCGCGGTGAGTT  GATGTAGGAGGGCGTGGATATGTCCT  GTATTGACCGATTCCTTGCGGTCCGAA  GGCTTGGCTGGAGCTAGTGGAGGTCAA  AACCCGCGGTCGGCATCTACTCTATTC  GACGTACCAGATTACGCTCAT-  -ATGTCGGAGCCATCATCAAA  TATCGATGCCCACCCGGGTGGAA-  -TTACATTGCGTGAGACACAA  CTGATCTCAGAGGAGGACCTGCAT-  -ATGTCGGAGCCATCATCAAA  CGCTGCAGGTCGACGGATCCCCGGGAA-  -TTACATTGCGTGAGACACAA  GACGTACCAGATTACGCTCAT-  -ATGGAACCTTTACGACTCAA  TATCGATGCCCACCCGGGTGGAA-  -TCACATCGCCAAATCGGCGA  CTGATCTCAGAGGAGGACCTGCAT-  -ATGGAACCTTTACGACTCAA  CGCTGCAGGTCGACGGATCCCCGGGAA-  -TCACATCGCCAAATCGGCGA  GACGTACCAGATTACGCTCAT-  -ATGGAGACGGAAATCCCCCC  TATCGATGCCCACCCGGGTGGAA-  -TTATACAGACTGATATAACA  CTGATCTCAGAGGAGGACCTGCAT-  -ATGGAGACGGAAATCCCCCC  CGCTGCAGGTCGACGGATCCCCGGGAA-  -TTATACAGACTGATATAACA  GTTGACAAACTCTCTTAGACC-  -GCCGAGATCCCAGATCGAA  TGGGATCTCGGCGGTCTA-  -AGAGAGTTTGTCAACATGTTGC  TTTGACCATCAGAGAATTCTTT-  -CCGATTTGAGCATCTCCC  GCTCAAATCGGAAAGAAT-  -TCTCTGATGGTCAAATACGTCG  AAAGTGGTCATACTTGATACCC-  -ACAAGAATAGGGATGGCC | *FgBUB2* deletion and southern probe  *FgBUB2* deletion  FgBub2-GFP complementation  *FgBFA1* deletion and southern probe  *FgBFA1* deletion  FgBfa1-GFP complementation  *FgBFA1* deletion  *FgBUB2*-*FgBFA1* double deletion  *FgTEM1* deletion and southern probe  FgTem1-GFP complementation  Yeast two hybrid  FgTem1 CA point mutation（Q163L）  FgTem1 DN point mutation（T118N） |
| *FgTRI1* QF | ACCAGGTCCTCAGTCTTG | qRT-PCR |
| *FgTRI1* QR | TCGTTGTGCTTGCCATAG |  |
| *FgTRI4* QF | TGAGGGATGTTGGATTGAGCAGTAC |  |
| *FgTRI4* QR | TGCTTCCGCTCATCAAACAGGT |  |
| *FgTRI5* QF | GCTACTCAGAATGCCCTCAG |  |
| *FgTRI5* QR | CGCATGTTATCCACCCTGCTA |  |
| *FgTRI6* QF | ATGATGATTGAGGATATGTTGT |  |
| *FgTRI6* QR | GAGTGATGAGGTCTGGAA |  |
| *FgTRI10* QF | GCTGTAACTGTCCCCAGCAT |  |
| *FgTRI10*QR | GTGAAGTTGCGACCGTACTC |  |
| *FgTRI12*QF | AGATCCGAGAACAAGCCTACTGC |  |
| *FgTRI12* QR | TTCCGCCTTGACCACCTTCATC |  |
| *FgActin* QF | ACGGAAACATTGTCATGTCTGGTG |  |
| *FgActin* QR | CTCTCGTCGTACTCCTGCTTGG |  |
| FgAlp6-mCherryF | Agggaacaaaagctgggtacc  TATACCTATTAGGAACTCGC | FgAlp6-mCherry |
| FgAlp6-mCherryR | Gcccttgctcaccataagctt  ACCCCTATTCGTCGAAACAC |  |
| FgBub2-ToxA-GF | GCATGGACGAGCTGTACAAG  ATGTCGGAGCCATCATCAAAC | ToxA-GFP-FgBub2 |
| FgBub2-ToxA-GR | CCCCCGGGCTGCAGGAATTC  ATTTGAGCTTCAACTTCCCT |  |
| FgBfa1-ToxA-GF | GCATGGACGAGCTGTACAAG  ATGGAACCTTTACGACTCAAA | ToxA-GFP-FgBfa1 |
| FgBfa1-ToxA-GR | CCCCCGGGCTGCAGGAATTC  CGGTGTCGTAACGCATAAA |  |
| ToxA-WF-Xho1: | GGGTACCGGGCCCCCCCTCGAG  TGGAATCCATGGAGGAGTTC | ToxA-sGFP |
| GFPR-TAA | CTTGTACAGCTCGTCCATGC |  |
| FgBub2-ZF-pCX62 | GAACAAAAGCTGGGT  CAACAACGGTGGAAAGGA | pFgBub2-Flag |
| FgBub2-OR-pCX62 | CAGCGGCGCGCCGAATTATTTGTCG  TCATCGTCTTTGTAGTCTTTGTCGT  CATCGTCTTTGTAGTCTTTGTCGTC  ATCGTCTTTGTAGTCCATACCAC  CCATTGCGTGAGACACAATCT |  |
| FgBub2-His-F | GCCATGGCTGATATCGGATCC  ATGTCGGAGCCATCATCAAA | His-FgBub2 |
| FgBub2-His-R | CTCGAGTGCGGCCGCAAGCTT  GTTACATTGCGTGAGACACAA |  |
| FgTem1-GST-F | GATCTGGTTCCGCGTGGATCC  ATGGAGACGGAAATCCCCCC | GST-FgTem1 |
| FgTem1-GST-R | TCGAGTCGACCCGGGAATTCCTT  ATACAGACTGATATAACA |  |
| Fg17139AR-G418 | AATGGAAATTGTAAGCGTTAATCTAGAGGTTGAGTCGTCCAGTTT | For double deletion of *FgTEM1* with *FgBUB2* or *FgTEM1* with *FgBFA1.* |
| Fg17139BF-G418 | TATCGCCTTCTTGACGAGTTCTTCTGAGACACGGCCATTTACGAG |  |
| G418F1 | TCTAGATTAACGCTTACAATTTCCA |  |
| G418R1 | GCCCAATAGCAGCCAGTCC |  |
| G418F2 | CAACAACACGCATCATCCCA |  |
| G418R2 | TCAGAAGAACTCGTCAAGAA |  |
| FgCdc10CF-GFP | agggaacaaaagctgggtaccCCACTAAGGCTCCATTCAC | FgCdc10-GFP |
| FgCdc10GR-GFP | gccgccgccgccgccaagcttGTAGCCGTTCATCGTCATTC |  |
| FgCdc11CF-GFP | agggaacaaaagctgggtaccCCAGGCGTGCCAATCTGCT | FgCdc11-GFP |
| FgCdc11CR-GFP | gccgccgccgccgccaagcttGTTGAGCTGCTCCTCGCCAC |  |
