# Supplementary figures and images for: The mitotic exit mediated by small GTPase Tem1 is essential for the pathogenicity of Fusarium graminearum
Source: PLoS Pathog. 2023 Mar 16;19(3):e1011255. doi: 10.1371/journal.ppat.1011255 (PMC10047555; doi:10.1371/journal.ppat.1011255)

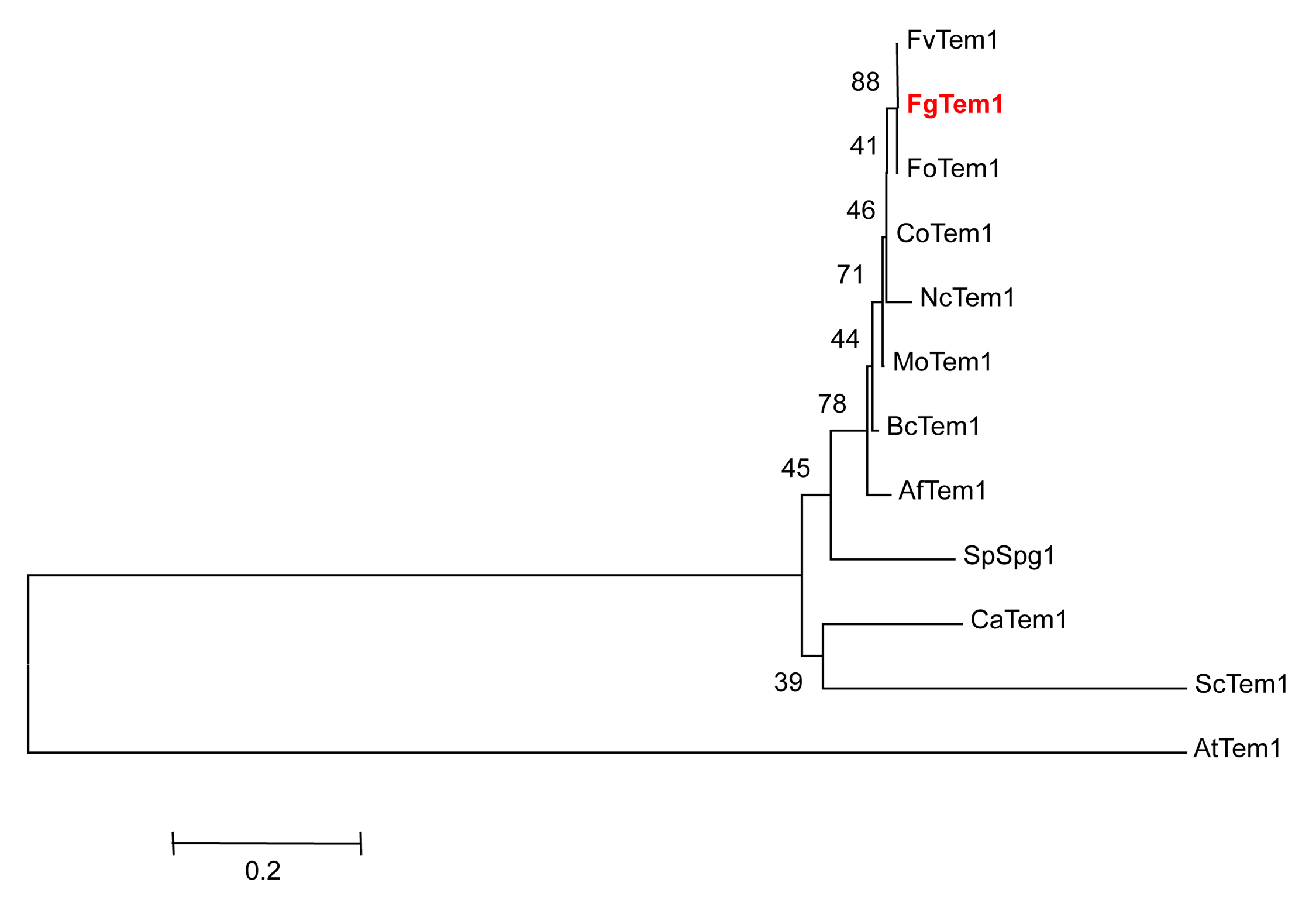

Supplement: S1 Fig — The phylogenetic analysis of Tem1 in F. graminearum is shown in relation to other fungal species. The sequence alignments were performed using the Clustal X 1.83 program and the phylogenetic tree was generated based on neighbor-joining method using MEGA 6.0 software with 10000 bootstrap replicates between Tem1 homologues in different organisms. Accession numbers: Aspergillus flavus (AfTem1-XP_002376572.1, AfBub2-RAQ49602.1, AfBfa1-XP_002382919.1), Botrytis cinerea (BcTem1-EMR82353.1, BcBub2-XP_001559021.1, BcBfa1-EMR88000.1), Candida albicans (CaTem1-XP_019330622.1, CaBub2-XP_715368.2, CaBfa1-RLP61346.1), Colletotrichum orbiculare (CoTem1-TDZ19252.1, CoBub2-TDZ14987.1, CoBfa1-TDZ23356.1), Fusarium graminearum (FgTem1-EYB30459.1, FgBub2-XP_011316822.1, FgBfa1-XP_011324890.1), Fusarium oxysporum (FoTem1-RKK77852.1, FoBub2-SCO79815.1, FoBfa1-RKK73093.1), Fusarium verticillioides (FvTem1-XP_018752224.1, FvBub2-XP_018743852.1, FvBfa1-RBR02439.1), Neurospora crassa (NcTem1-XP_960671.3, NcBub2-XP_965337.1, NcBfa1-KHE87150.1), Magnaporthe oryzae (MoTem1-XP_018752224.1, MoBub2-XP_003710862.1, MoBfa1-XP_003716829.1), Saccharomyces cerevisiae (ScTem1-QHB10674.1, ScBub2-ONH76596.1, ScBfa1-AJR73492.1), Ustilago maydis (UmBub2-XP_011386392.1, UmBfa1-XP_011389318.1), Schizosaccharomyces pombe (SpSpg1-NP_593285.1, SpBub2-NP_593901.1, SpBfa1-NP_593149.1), Arabidopsis thaliana (AtTem1-OAP14749.1). (TIF) [file ppat.1011255.s004.tif]

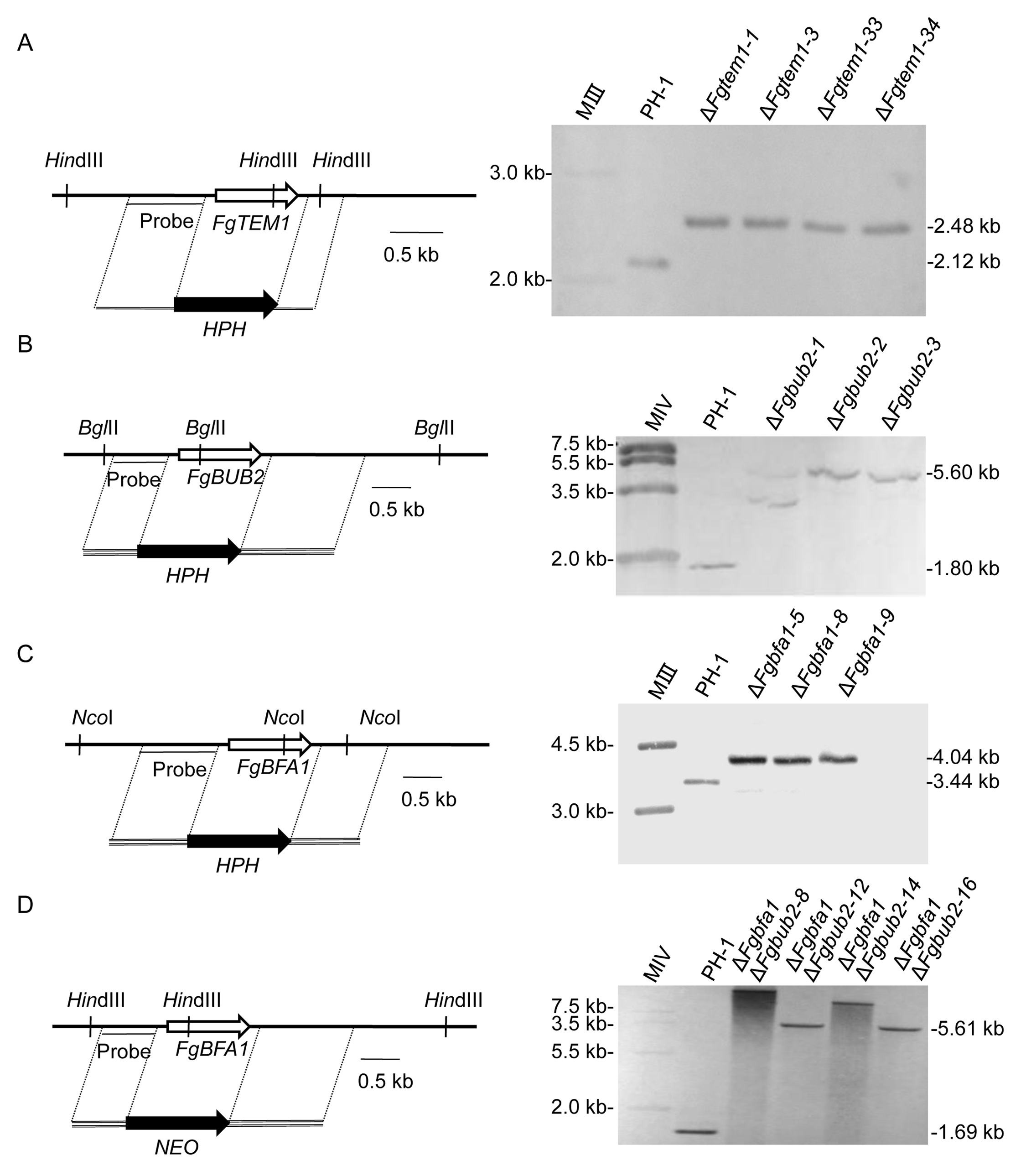

Supplement: S2 Fig — (A) The scheme for split-marker approach based on the targeted gene replacement of FgTEM1 with hygromycin resistance (HPH) gene. Genomic DNAs were extracted from PH-1 and the putative transformants. Hind III-digested DNAs showed a 2.12 kb band in the PH-1 and a 2.48 kb band in the mutants. (B) The scheme for split-marker approach based on the targeted gene replacement of FgBUB2 with HPH gene. Genomic DNAs were extracted from PH-1 and the putative transformants. BgI II-digested DNAs showed a 1.80 kb band in the PH-1 and a 5.60 kb band in the mutants. (C) The scheme for split-marker approach based on the targeted gene replacement of FgBFA1 with HPH gene. Genomic DNAs were extracted from PH-1 and the putative transformants. Nco I-digested DNAs showed a 3.44 kb band in the PH-1 and a 4.04 kb band in the mutants. (D) The scheme for split-marker approach based on the targeted gene replacement of FgBFA1 with NEO gene. Genomic DNAs were extracted from PH-1 and the putative transformants. Hind III-digested DNAs showed a 1.69 kb band in the PH-1 and a 5.61 kb band in the mutants. (TIF) [file ppat.1011255.s005.tif]

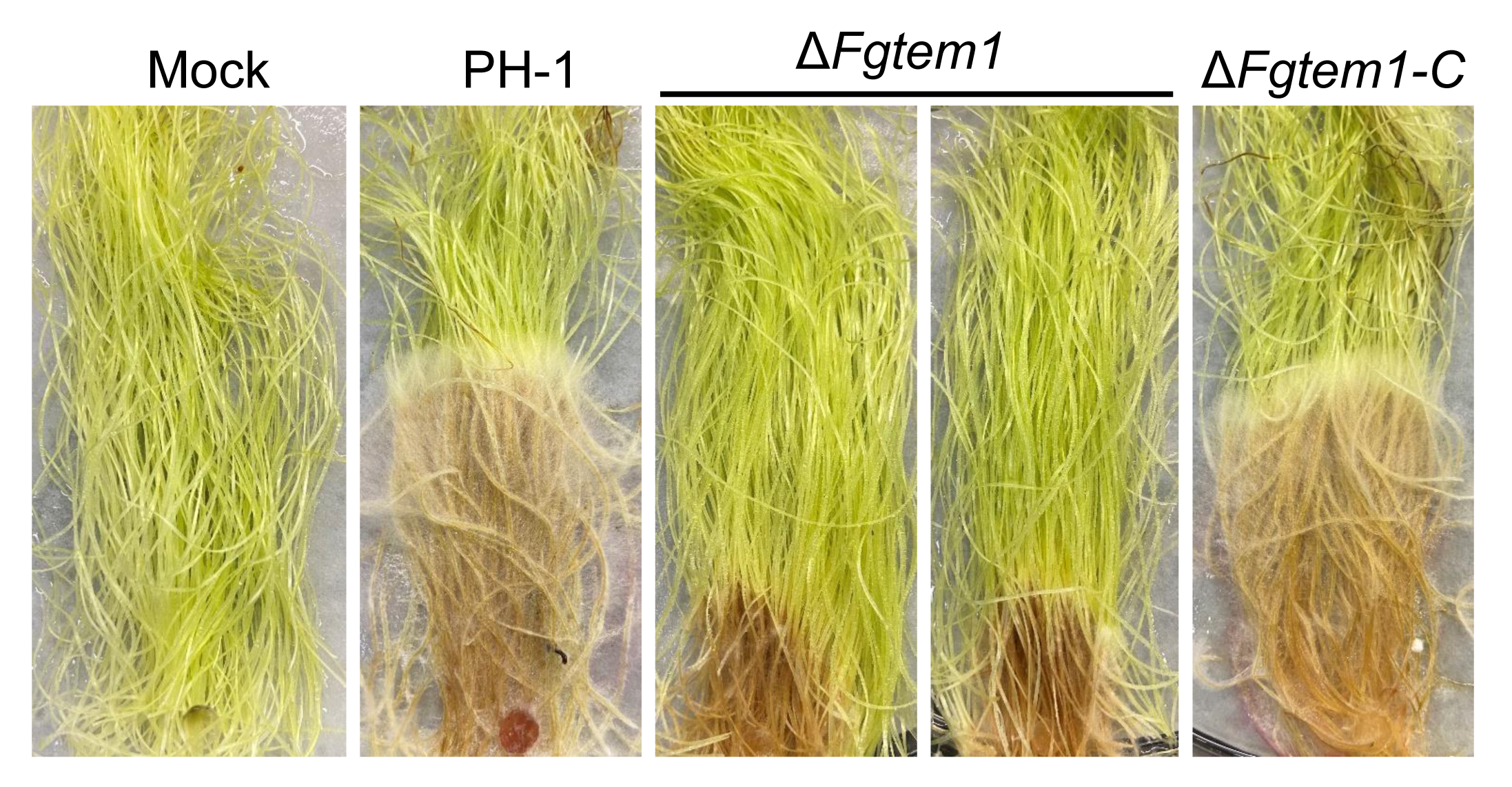

Supplement: S3 Fig — A small block of SYM agar cultures of wild type PH-1, ΔFgtem1 and ΔFgtem1-C strains were placed on freshly corn silks for 7 days. ΔFgtem1 mutant significantly reduced the pathogenicity. (TIF) [file ppat.1011255.s006.tif]

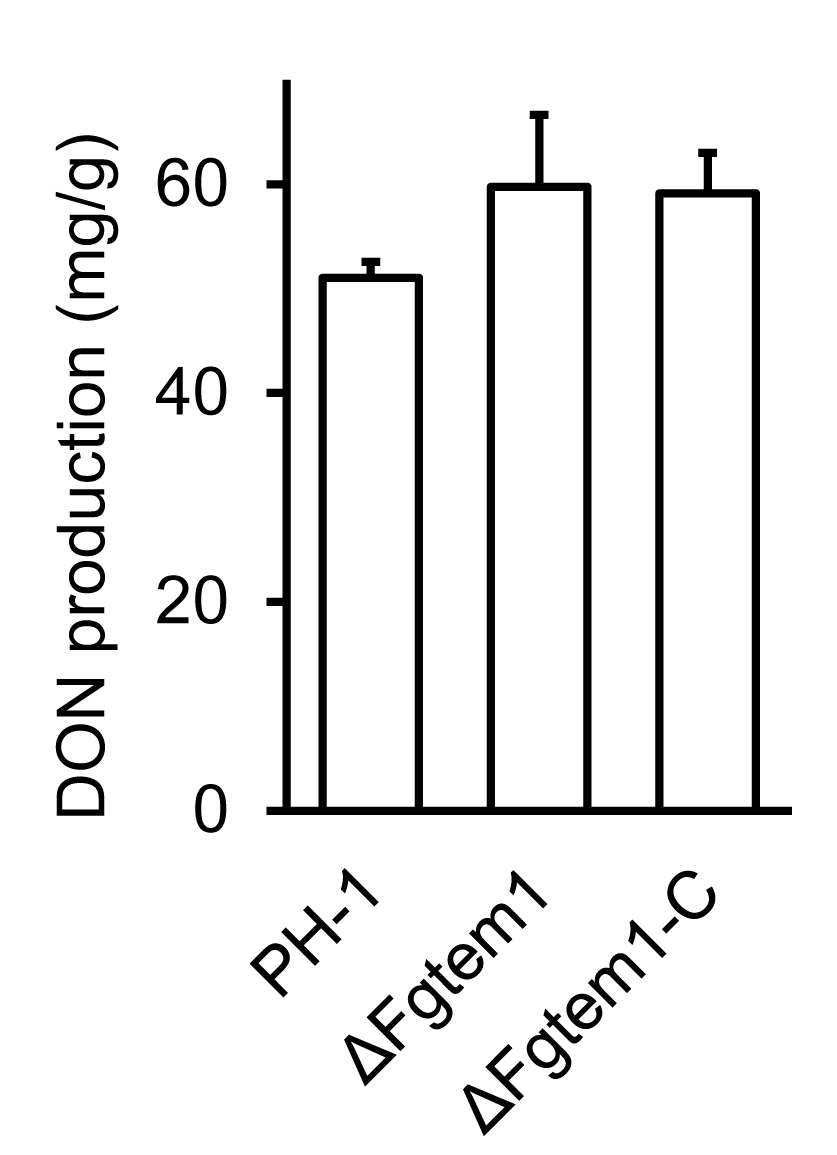

Supplement: S4 Fig — Two-tailed Student t-test was used for paired comparison of the DON produced by ΔFgtem1 and PH-1. (P = 0.16, no significant difference). (TIF) [file ppat.1011255.s007.tif]

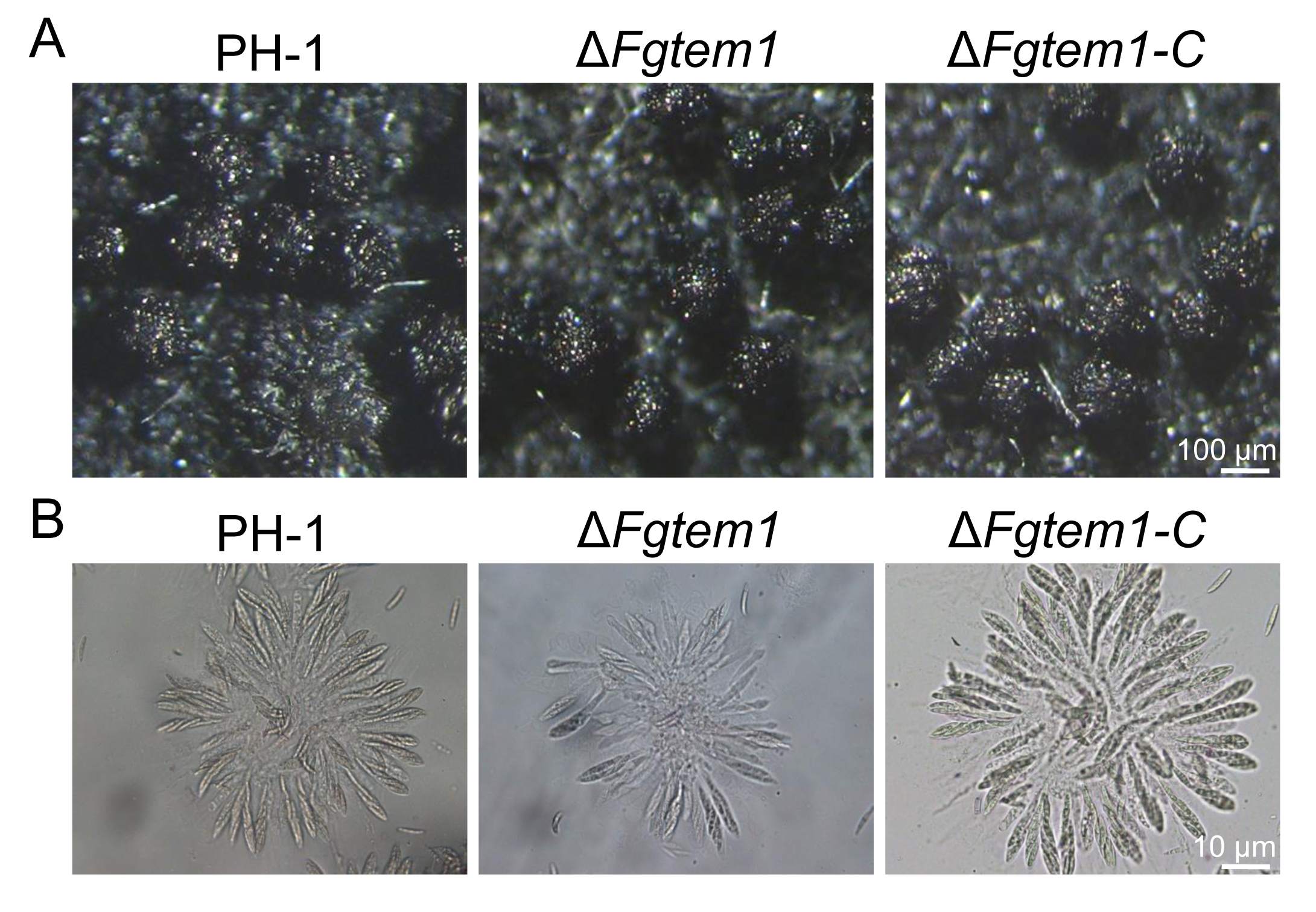

Supplement: S5 Fig — (A) Perithecia formation of the wild type PH-1, ΔFgtem1 and ΔFgtem1-C strains on carrot agar plates after 9 days. (B) The ascospore released from the perithecia of the indicated strains on carrot agar plates after 9 days. (TIF) [file ppat.1011255.s008.tif]

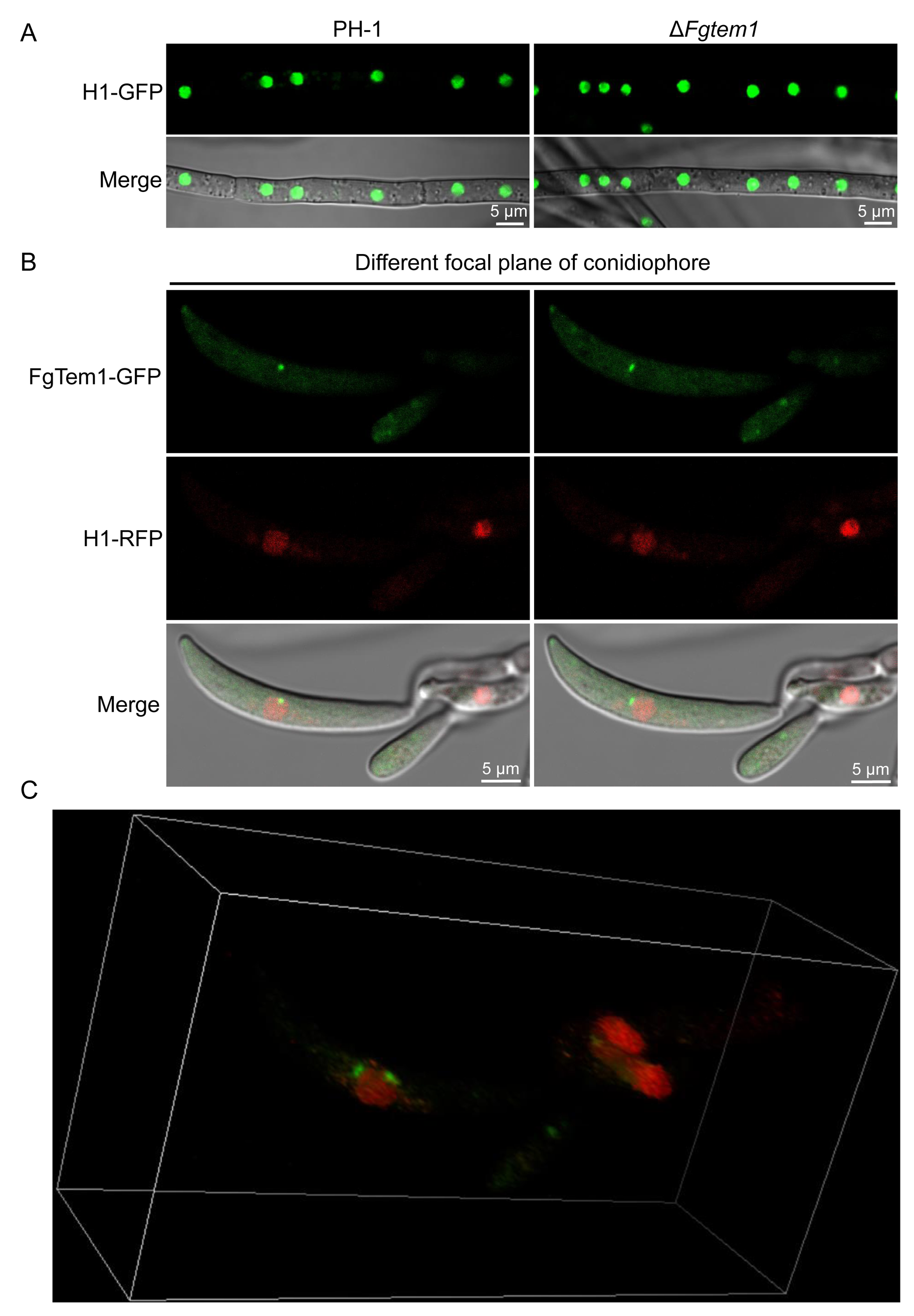

Supplement: S6 Fig — (A) The distribution of nuclei (H1-GFP) in the hyphae of the PH-1 and ΔFgtem1. (B) FgTem1-GFP localizes to the periphery of nuclei in different focal planes of conidiophore. (C) 3-D (three-dimensional) micrograph showing FgTem1-GFP close to nuclear periphery. (TIF) [file ppat.1011255.s009.tif]

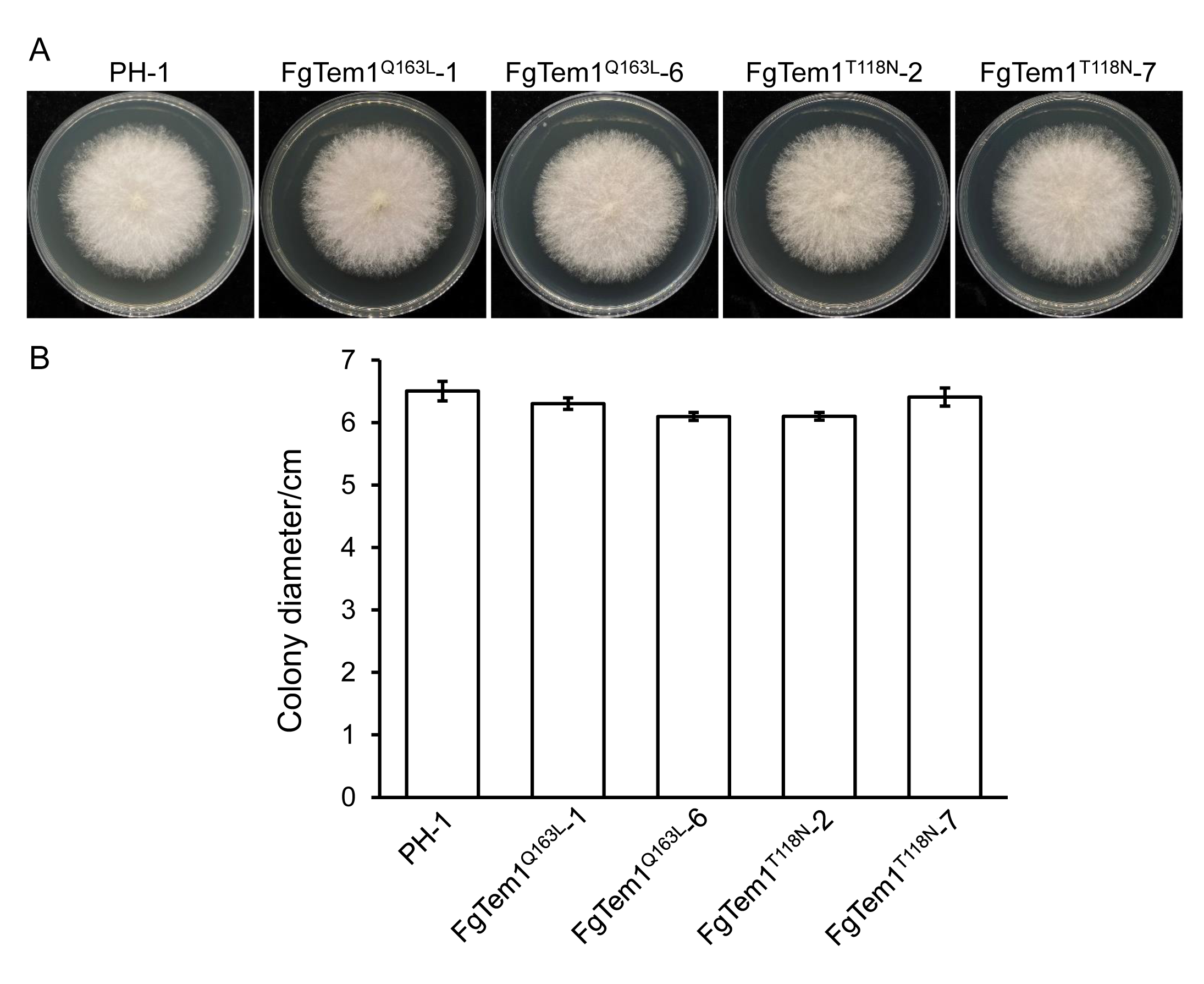

Supplement: S7 Fig — (A-B) Colony morphologies and diameters of the wild type (PH-1), constitutive activate (FgTem1Q163L) and dominant negative (FgTem1T118N) isoforms of FgTem1 strains grown on CM at 28°C for 3 days. (TIF) [file ppat.1011255.s010.tif]

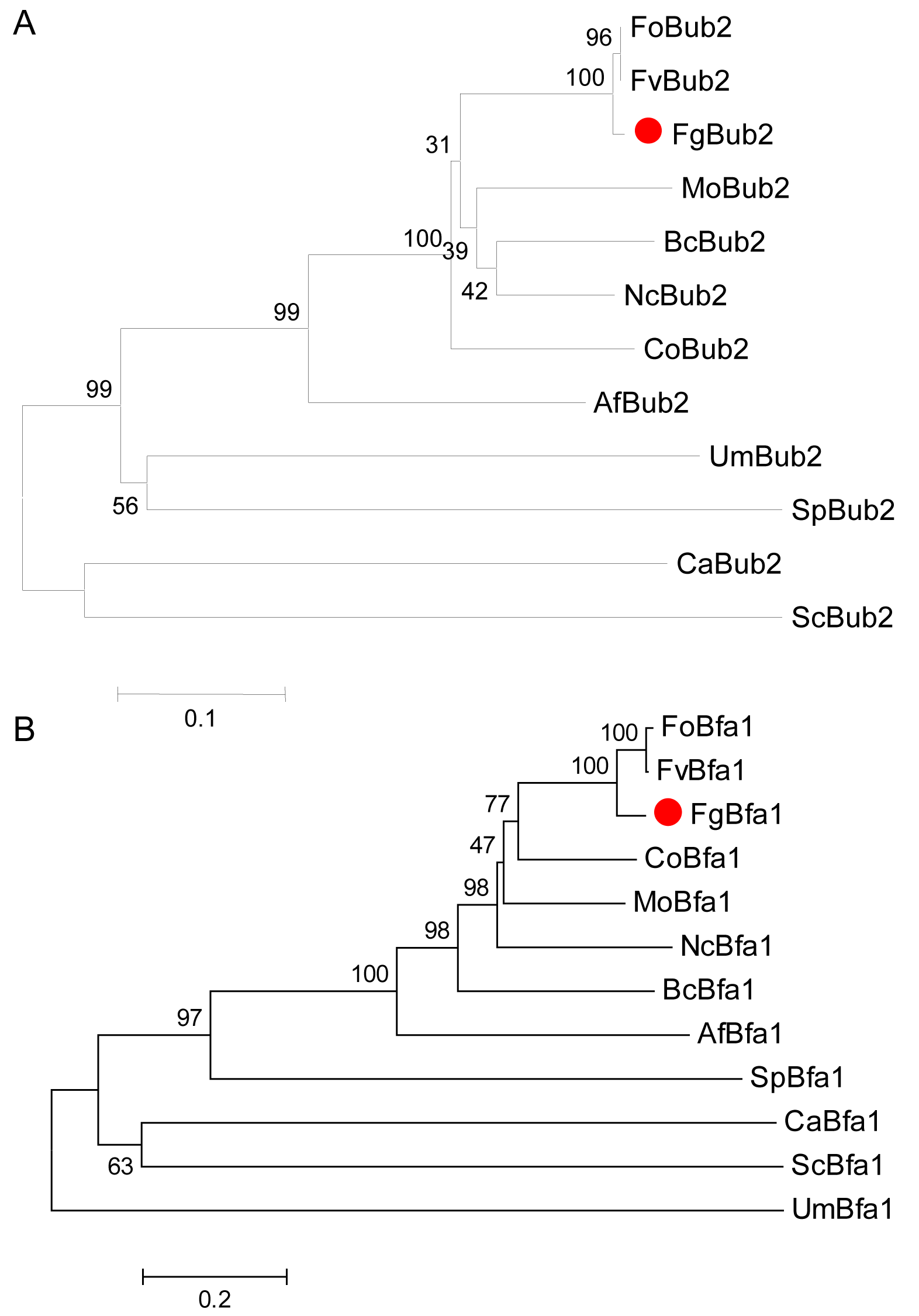

Supplement: S8 Fig — (A-B) Phylogenetic analyses of FgBub2 and FgBfa1 with other fungal species. Sequence alignments were performed using Clustal X 1.83 program and the phylogenetic tree was generated based on neighbor-joining method using MEGA 6.0 software with 10000 bootstrap replicates between Bub2 and Bfa1 homologues in different organisms. (TIF) [file ppat.1011255.s011.tif]

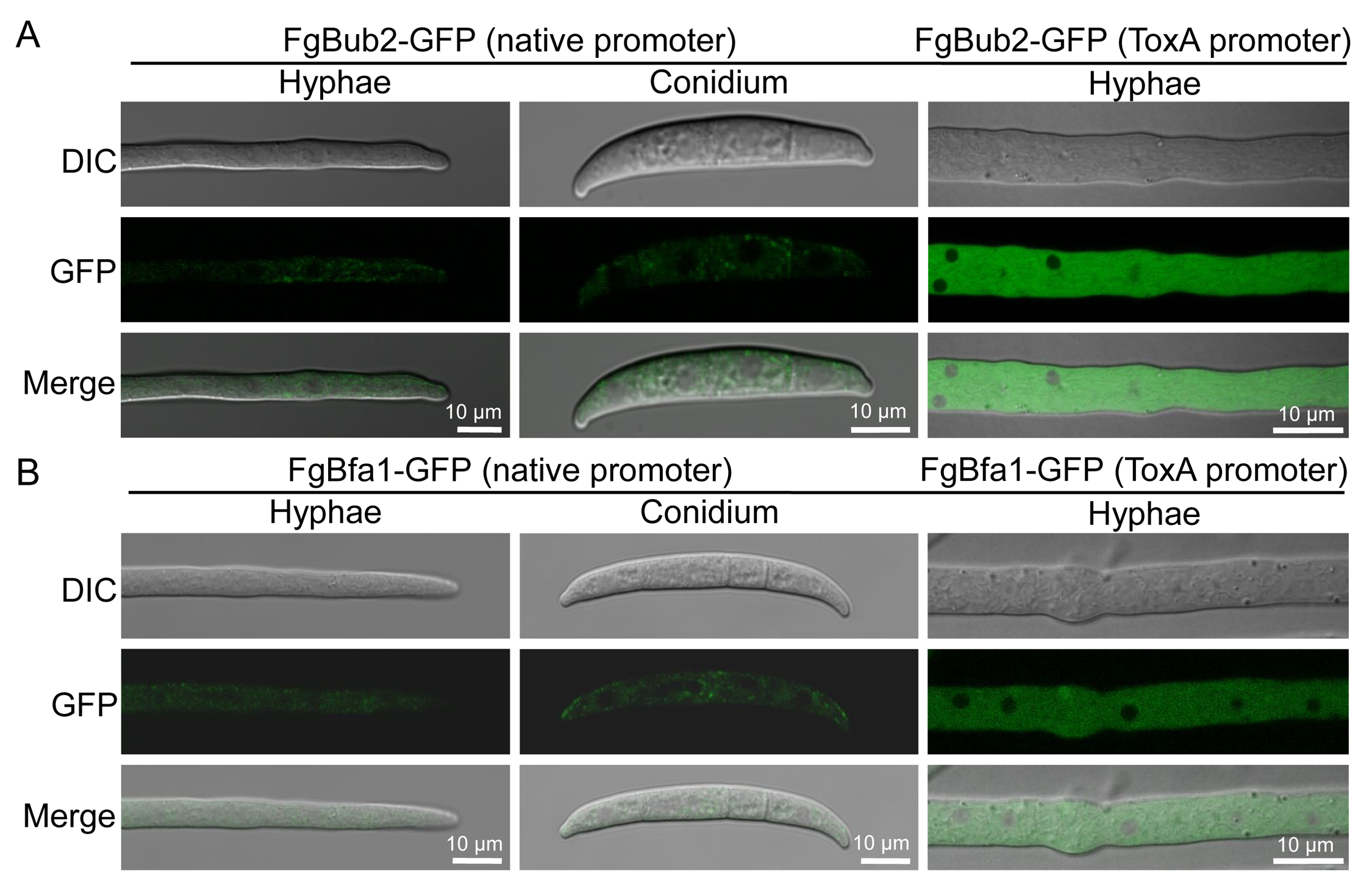

Supplement: S9 Fig — (A) FgBub2-GFP is diffuse in the cytoplasm under the native and ToxA promoters. (B) FgBfa1-GFP is diffuse in the cytoplasm under the native and ToxA promoters. (TIF) [file ppat.1011255.s012.tif]
